# A Multimethodological Approach for the Chemical Characterization of Edible Insects: The Case Study of *Acheta domesticus*

**DOI:** 10.3390/foods12122331

**Published:** 2023-06-09

**Authors:** Mattia Spano, Giacomo Di Matteo, Carlos Alberto Fernandez Retamozo, Alba Lasalvia, Marco Ruggeri, Giuseppina Sandri, Carlos Cordeiro, Marta Sousa Silva, Carlotta Totaro Fila, Stefania Garzoli, Maria Elisa Crestoni, Luisa Mannina

**Affiliations:** 1 Department of Chemistry and Technology of Drugs, Sapienza University of Rome, Piazzale Aldo Moro 5, 00185 Rome, Italy; mattia.spano@uniroma1.it (M.S.); giacomo.dimatteo@uniroma1.it (G.D.M.); carlosalbertostefano.fernandez@gmail.com (C.A.F.R.); alba.lasalvia@uniroma1.it (A.L.); stefania.garzoli@uniroma1.it (S.G.); mariaelisa.crestoni@uniroma1.it (M.E.C.); 2 NMR-Based Metabolomics Laboratory (NMLab), Sapienza University of Rome, Piazzale Aldo Moro 5, 00185 Rome, Italy; 3 Department of Drug Sciences, University of Pavia, Viale Taramelli 12, 27100 Pavia, Italy; marco.ruggeri02@universitadipavia.it (M.R.); g.sandri@unipv.it (G.S.); 4 Laboratório de FT-ICR e Espectrometria de Massa Estrutural, Faculdade de Ciências, Universidade de Lisboa, Campo-Grande, 1749-016 Lisboa, Portugal; cacordeiro@ciencias.ulisboa.pt (C.C.); mfsilva@ciencias.ulisboa.pt (M.S.S.); 5 Alia Insect Farm, Via Olmetto, 20123 Milan, Italy; carlotta@aliainsectfarm.it

**Keywords:** *A. domesticus* powder, chemical characterization, NMR spectroscopy, FT-ICR MS, SPME-GC-MS

## Abstract

*Acheta domesticus* (house cricket) has been recently introduced into the official European list of novel foods, representing an alternative and sustainable food source. Up to now, the chemical characterization of this edible insect has been focused only on specific classes of compounds. Here, three production batches of an *A. domesticus* powder were investigated by means of a multimethodological approach based on NMR, FT-ICR MS, and GC-MS methodologies. The applied analytical protocol, proposed for the first time in the study of an edible insect, allowed us to identify and quantify compounds not previously reported in crickets. In particular, methyl-branched hydrocarbons, previously identified in other insects, together with other compounds such as citrulline, formate, *γ*-terpinene, *p*-cymene, *α*-thujene, *β*-thujene, and 4-carene were detected. Amino acids, organic acids, and fatty acids were also identified and quantified. The improved knowledge of the chemical profile of this novel food opens new horizons both for the use of crickets as a food ingredient and for the use of extracts for the production of new formulations. In order to achieve this objective, studies regarding safety, biological activity, bioaccessibility, and bioavailability are needed as future perspectives in this field.

## 1. Introduction

There is an ever-growing need for research into new and innovative food sources, considering the expected large population increase and the necessity of reducing the ecological problems related to intensive food production [1]. The importance of this focus has also been highlighted in the United Nations 2030 Agenda, whose goals include zero hunger, good health and well-being, responsible consumption and production, climate action, and life on land [2]. In this context, edible insects represent an important potential food source capable of satisfying both sustainability and nutritional demands. Several sustainability advantages have been related to edible insect production, such as low water consumption, with an average expenditure of 1 L/Kg of insect proteins respect to 1.500 L/Kg of cattle proteins, lower feed consumption per Kg of obtained proteins, the generation of less greenhouse gases and ammonia, and the need for smaller spaces for farming [3,4]. At the same time, edible insects are a good source of many essential nutrients such as proteins, fatty acids, minerals, and, in some cases, vitamins [5]. Entomophagy is a common practice in several cultures such as in Latin America, Asia, and Africa, with beetles, caterpillars, bees, wasps, and ants being the most-consumed insects [6]. Conversely, in Europe, the use of edible insects as food represents a niche practice. Nevertheless, in recent years, the environmental and nutritional advantages of edible insects have encouraged their gradual recognition as novel foods. Among them, *Acheta domesticus* (house cricket) represents the last edible insect added to the official European list of novel foods, in frozen, dried and powder forms, through the Commission Implementing Regulation (EU) 2022/188 of 10 February 2022 [7]. To date, its chemical investigations reported in the literature have been focused on specific classes of compounds determined using appropriate targeted methodologies. The amino acid profile of cricket powder [8,9,10] as well as the fatty acid contents of cricket extracts [8,9,10,11,12] and their elemental profile [9,12] have been reported. Moreover, phenolic acids and flavonoids have been measured in organic and commercial *A. domesticus* [13].

Considering the increasing interest in and use of this edible insect, it is important to further develop the knowledge regarding its chemical composition. For this purpose, the simultaneous application of several advanced methodologies could be an effective strategy, as demonstrated in previous studies [14,15].

On the basis of these premises, in the present study, a spray-dried *A. domesticus* powder was investigated, for the first time, through a multimethodological approach including both untargeted nuclear magnetic resonance (NMR) spectroscopy and Fourier transform ion cyclotron resonance mass spectrometry (FT-ICR MS) and targeted gas chromatography–mass spectrometry (GC-MS) analyses to obtain a comprehensive chemical profile. In particular, the NMR and FT-ICR MS methodologies are recognized as the two main powerful untargeted approaches to achieve the metabolomic profiling of natural matrix extracts [16], since these techniques are able to identify, with a single analysis, several classes of compound present in a complex matrix. On the other hand, GC-MS is a targeted methodology useful for identifying and quantifying specific classes of compounds. Altogether, these complementary methodologies can be useful for obtaining the most co-exhaustive chemical profile of the product possible.

## 2. Materials and Methods

### 2.1. Sampling

In order to preserve the chemical composition of crickets and obtain a homogeneous fine powder to optimize the extractions, a production approach previously described [17], based on spray-drying, was used. Spray-drying is a pulverization approach that has been shown to be efficient and appropriate when applied to edible insects [18]. This drying method is characterized by a very short heat exposure time (a few seconds) with respect to the classic oven-drying method, which is several hours long. As a consequence, the Maillard reaction is strongly reduced, as are the “roasted” color and flavor typical of oven-dried insects. For powder production, insects 35–42 days old (one or two stages before the complete adult stage) were pasteurized and then mixed into the same volume of water to obtain a homogeneous slurry after mining and milling. The mixture was spray-dried at 200 °C (inlet temperature), producing a fine powder. The powder was preserved in sealed bag kept away from light and heat sources until analysis. Three batches were produced, and 50 g from each one was sampled and analyzed.

### 2.2. Chemicals and Reagents

Gradient-grade water, chloroform, and methanol were purchased from Merck Life Science (Milano, Italy). Deuterated water (D_2_O), 99.97 atom% of deuterium, and 3-(trimethylsilyl)-propionic-2,2,3,3-d4 acid sodium salt (TSP) were purchased from Euriso-Top (Saclay, France). Potassium phosphate monobasic (KH_2_PO_4_) and potassium phosphate dibasic (K_2_HPO_4_) were purchased from Merck Life Science (Milano, Italy).

### 2.3. NMR Analysis

Sample extraction for NMR analysis was carried out by modifying a previously described protocol [19]. In detail, 100 mg of the sample was added to 3 mL of a CH_3_OH/CHCl_3_ 2:1 *v*/*v* mixture and 0.8 mL of bidistilled water. The obtained system was sonicated at room temperature for 10 min, followed by the addition of 1 mL of chloroform and 1 mL of bidistilled water. The hydroalcoholic phase was finally separated after centrifugation for 15 min (25 °C, 7200× *g*) and dried with a soft N_2_ flux. The entire procedure was repeated two more times on the sample residue, guaranteeing a quantitative metabolite extraction.

The dried hydroalcoholic phase was dissolved in 700 μL of 200 mM phosphate buffer/D_2_O containing 0.4 mM TSP (3-(trimethylsilyl)propionic acid sodium salt) as the internal standard for metabolite quantification. NMR analyses were carried out on a Jeol JNM-ECZ 600R (JEOL Ltd., Tokyo, Japan) operating at a proton frequency of 600.17 MHz and equipped with a Jeol 5 mm FG/RO DIGITAL AUTOTUNE probe. ^1^H NMR experiments were carried out using the following parameters: 298 K, 128 scans, residual water signal suppression with a presaturation pulse, 7.7 s relaxation delay, 90° pulse of 8.3 μs, 64 K data points, and 9000 Hz spectral width. ^1^H spectra were referenced to methyl group signals of TSP (δ_H_ = 0.00 ppm) in D_2_O. A homonuclear ^1^H-^1^H TOCSY experiment was carried out with 52 scans, 8 K data points in *f*_2_ and 128 in *f*_1_, 50 ms mixing time, 2 s relaxation delay, and 9000 Hz spectral width in both dimensions. A heteronuclear ^1^H-^13^C HSQC experiment was carried out with 76 scans, 8 K data points in *f*_2_ and 256 in *f*_1_, 3 s relaxation delay, and a spectral width of 9000 Hz and 33,000 Hz for *f*_2_ and *f*_1_, respectively. A heteronuclear ^1^H-^13^C HMBC experiment was carried out with 64 scans, 8 K data points in *f*_2_ and 165 in *f*_1_, 2 s relaxation delay, and a spectral width of 9000 Hz and 37,500 Hz for *f*_2_ and *f*_1_, respectively Spectrum processing and signal integration were carried out with JEOL Delta software (v5.3.1). For metabolite quantification, the integrals of the corresponding selected resonances in the ^1^H-NMR spectra were measured with respect to TSP. Three replicates were made for each batch, and the results are expressed as mg/100 g of sample ± SD by applying the following equations:

C_X_ = (I_X_/I_TSP_) * (N_TSP_/N_X_) * C_TSP_ (1)

P_X_ = C_X_ * V * MW_X_ (2)

where C_X_ is the mM concentration of the quantified metabolite, I_X_ is the area of the metabolite integrated signal, I_TSP_ is the area of the internal standard T_SP_ signal, N_TSP_ is the proton number corresponding to the internal standard T_SP_ signal, N_X_ is the proton number corresponding to the metabolite signal, C_TSP_ is the mM concentration of the internal standard TSP, P_X_ is the mg amount of the metabolite in 100 mg of the sample, V is the volume of deuterated solvent used to solubilize the dried extract, and MW_X_ is the molecular weight of the selected metabolite.

### 2.4. FT-ICR MS Analysis

Stock solutions (1 mg/mL) of Bligh–Dyer hydroalcoholic and organic extracts of cricket powder were filtered through 0.45 μm hydrophobic polypropylene Acrodisc (Sigma-Aldrich, St. Louis, MO, USA) to eliminate debris and then diluted to a final concentration of 0.1–0.2 mg/L in methanol. Formic acid (1% *v*/*v*) and ammonia solution (24% *v*/*v*, 2 µL) were added to all replicates to assist (de)protonation in the positive (ESI (+)) and negative (ESI (−)) polarity mode analyses, respectively, whereas 0.5 μg L^−1^ leucine enkephalin (YGGFL, C_28_H_37_N_5_O_7_) was used as an internal calibrant revealed at *m*/*z* 556.27657 (M^+^H^+^). Further assessment of precision and mass accuracy was achieved by referring to ubiquitous metabolites, such as amino acids and fatty acids, so achieving a maximum mass deviation lower than 0.1 ppm. For each extract, three solutions were prepared as described above and directly infused with a flow rate of 120 mL h^−1^ in an electrospray ionization (ESI) source.

Preliminary ESI (+) MS analyses were performed with a Bruker BioApex Fourier transform ion cyclotron resonance (FT-ICR) mass spectrometer (Bruker Daltonics GmbH, Bremen, Germany) coupled with an Apollo I ESI source and a 4.7 T superconducting magnet (FT-ICR lab, Sapienza Università di Roma). Complementary information for the determination of fatty acids was gathered by using ESI (−) coupled to a linear ion trap mass spectrometer (LTQ XL, Thermo Fisher Scientific, Waltham, MA, USA). Ultrahigh-resolution mass analyses were carried out on a Bruker SolariX XR FT-ICR equipped with a 7 T superconducting magnet (Magnex Scientific Inc., Yarnton, UK), a ParaCell (Bruker Daltonics GmbH, Bremen, Germany), and an APOLLO II ESI source operated at the Universidade de Lisboa. Spectral analyses, compound identification, and annotation were achieved as already reported [20]. MS spectra were acquired in absorption mode considering a mass range of 100–1000 (resolution of 650,000 at *m*/*z* 400), whereas 200 scans with an acquisition size of 4M were co-added to collect the time domain data. Analyses were carried out in triplicate for each batch.

### 2.5. SPME-GC-MS Analysis of Volatile Compounds

To characterize the volatile chemical profile of *A. domesticus* powder, sampling was carried out using the SPME technique. A small amount of powder (~1 g) was placed in a 7 mL glass vial with a PTFE-coated silicone septum. With the aim of extracting the volatile compounds, a DVB/CAR/PDMS (divinylbenzene/carboxen/polydimethylsiloxane) SPME device was used.

The fiber was conditioned at 270 °C for 30 min. Sample equilibration was carried out at 40 °C for 20 min before sampling. The fiber was then exposed to the headspace of the sample for 30 min at 40 °C and inserted into the GC injector at 250 °C in splitless mode.

The gas chromatographic analyses were carried out on a Clarus 500 model Perkin Elmer (Waltham, MA, USA) coupled with a mass spectrometer equipped with an FID detector and a Varian Factor Four VF-1 capillary column. The temperature of the oven was initially programmed at 60 °C, increased to 220 °C with a rate of 6°/min, and maintained for 15 min. A carrier helium flow of 1 mL/min was used. Mass acquisitions were carried out at 70 eV (EI) in scan mode, considering a 40–400 *m*/*z* range at 220 °C.

The Wiley 2.2 and Nist 02 mass spectra libraries were used for volatile compound identification, together with the linear retention index (LRIs) calculation using alkane standards analyzed in the same conditions. The relative concentrations, expressed as percentage, were calculated using the FID signal peak areas. For each batch, analyses were carried out in triplicate.

### 2.6. GC-MS Analysis of Hexane Extract

Apolar fraction was extracted by adding 3 mL of hexane to 100 mg of the sample, leaving the system under stirring for 3 h. After extraction, 1 µL of extract was manually injected at 270 °C using a split ratio of 1:20. Analyses started at 60 °C, reaching 170 °C at 4°/min followed by an increase to 250 °C for 3 min at a rate of 5°/min and finally being maintained for 15 min. The mass spectrometer operated in the same conditions reported in Section 2.5, as well as metabolite identification and quantification. Analyses were carried out in triplicate for each batch.

### 2.7. GC-MS Determination of Fatty Acid Content

FA content was determined after a derivatization process [21]. GC-MS analyses were carried out on the same apparatus reported above. In this case, the GC oven was equipped with a Restek Stabilwax polar capillary column. A carrier helium flow of 1 mL/min was used. The injector temperature was set at 280 °C and the oven temperature was programmed from 170 °C at a rate of 3 °C/min to 260 °C for 10 min. A volume of 2 µL was injected into the column in splitless mode; the mass spectra were recorded at 70 eV (EI) and were scanned in the range of 40–500 m/z. The ion source and the connection parts’ temperatures were 220 °C.

The mass spectrometer operated in the same conditions reported in Section 2.5, as well as metabolite identification and quantification. Analyses were carried out in triplicate for each batch.

## 3. Results

A metabolomics investigation of the analyzed Acheta domesticus powder is described here, reporting the results obtained using each methodology (NMR, FT-ICR MS, GC-MS) and then discussing the metabolite profile for the class of compounds.

### 3.1. NMR Analysis

**Figure 1:**
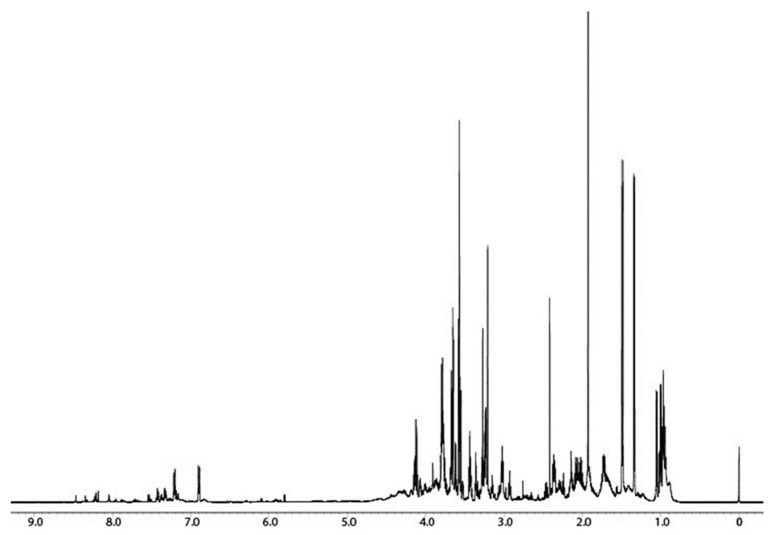
The 600 MHz ^1^H NMR spectrum of *A. domesticus* hydroalcoholic extract in a 200 mM phosphate buffer (pH 7.4)/D_2_O mixture with 0.4 mM of 3-(trimethylsilyl)-propionic-2,2,3,3-d4 acid sodium salt (TSP).

The ^1^H NMR spectrum of the hydroalcoholic extracts of cricket powder is presented in Figure 1. The metabolite assignment was obtained by means of 2D experiments and literature data regarding other biological matrices analyzed in the same NMR experimental conditions [14,19]. Moreover, when spin correlations in the 2D experiments were not adequate for confirming the presence of the metabolites, standard compound addition was carried out.

The following compounds, reported in Table 1, were assigned and quantified: nineteen amino acids and derivatives (alanine, aspartate, betaine, citrulline, glycine, glutamate, glutamine, histidine, isoleucine, leucine, lysine, methionine, phenylalanine, proline, taurine, threonine, tryptophan, tyrosine, valine), five organic acids (acetate, formate, fumarate, lactate, succinate), and other compounds, namely choline and glycerol.

### 3.2. FT-ICR MS Analysis

**Figure 2:**
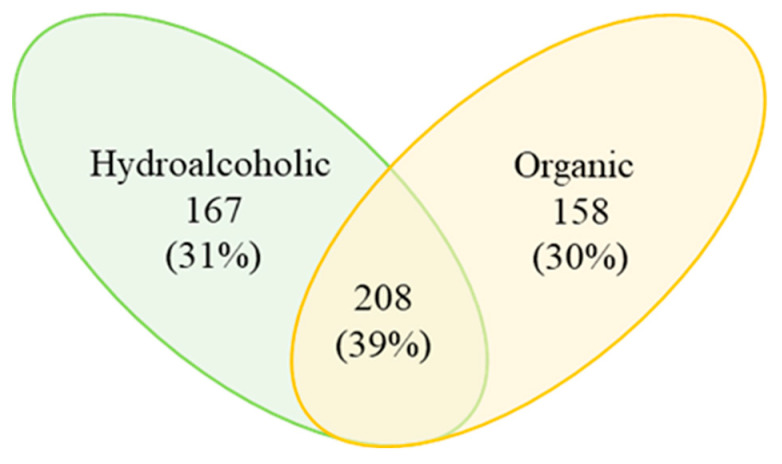
Metabolite distribution in hydroalcoholic (green) and organic (yellow) extracts shown in a two-way Venn diagram. The results are reported as absolute numbers of total hits.

FT-ICR MS analysis applied to hydroalcoholic and organic Bligh–Dyer extracts allowed us to simultaneously detect many compounds based on their accurate mass and specific isotope pattern. Different metabolomic databases enabled the identification of more than 500 molecular formulas, as shown in Table S1, spread between hydroalcoholic and organic Bligh–Dyer extracts. Among these, about 350 molecular formulas were attributed using the free tool MassTRIX [22]. Similarities and differences between the hydroalcoholic and organic extracts are underlined in a two-way Venn diagram; around 39% of the molecular formulas were revealed to be common to both hydroalcoholic and organic extracts, as shown in Figure 2.

**Figure 3:**
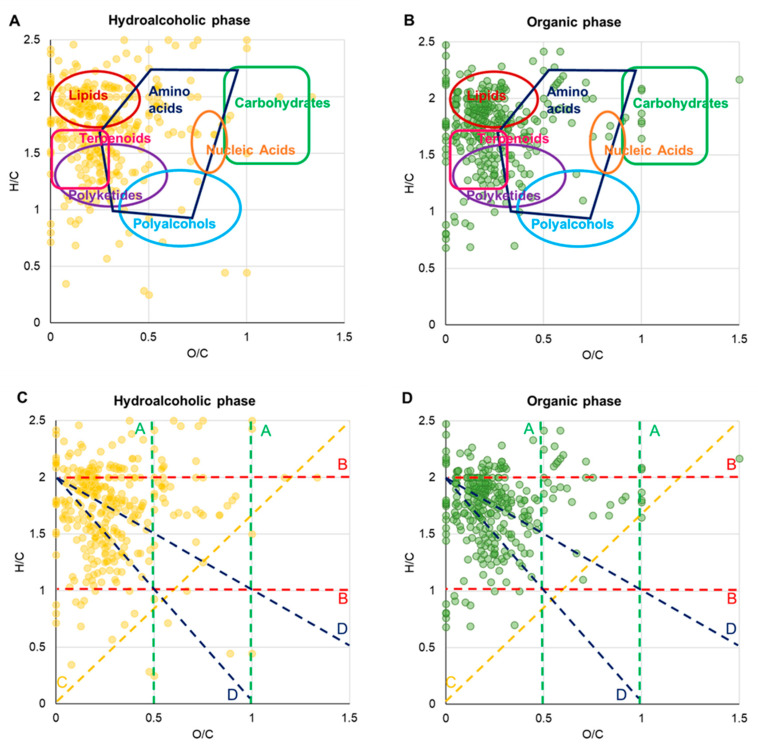
van Krevelen diagrams (elemental plots) obtained from the molecular formulas achieved through ESI FT-ICR MS analysis of hydroalcoholic (panel (**A**)) and organic phases (panel (**B**)) of cricket powder. Homology series along dashed lines, related to the following chemical reactions: (de)hydrogenation (A lines); oxidation or reduction (B lines); (de)hydration and condensation processes (C lines); (de)methylation (D lines), are displayed in panels (**C**,**D**) (hydroalcoholic and organic extracts, respectively).

Considering the high number of molecular formulas, van Krevelen diagrams (vKds) were built to provide a visual distribution of the main identified molecular classes [23]. In particular, elemental formulas from each sample were inserted into the diagrams, where the molar hydrogen to carbon ratio (H/C) is plotted against the molar ratio of oxygen to carbon (O/C), thus affording an overview of the molecular families. The hydroalcoholic extract presented a high density of metabolites in the region of lipids, terpenoids, and polyketides, followed by amino acids, whereas the organic portion displays a higher number of entries in the lipid and terpenoid areas, as shown in Figure 3A,B.

Along the trend lines in the corresponding diagrams in Figure 3C,D, it is possible to identify structural relationships due to chemical reactions among groups of compounds. For instance, hits along vertical green A lines, associated with (de)hydrogenation processes, comprise decenedioic/sebacic acids (C_10_H_16_O_4_/C_10_H_18_O_4_), linoleamide/oleamide/stereamide (C_18_H_33_NO/C_18_H_35_NO/C_18_H_37_NO), and hydroxy-octadecenoylcarnitine/hydroxy-octadecanoylcarnitine (C_25_H_47_NO_5_/C_25_H_49_NO_5_). Items along horizontal red B lines, related to oxidation and reduction reactions, include vitamin D3/calcidiol/calcitriol (C_27_H_44_O/C_27_H_44_O_2_/C_27_H_44_O_3_) and palmitoleic/keto-palmitic acids (C_16_H_30_O_2_/C_16_H_30_O_3_). Entries along bisector yellow C lines, due to hydration and condensation paths, encompass α-tocopheronolactone/α-tocopheronic acid (C_16_H_22_O_4_/C_16_H_24_O_5_). Peaks on blue D lines with intercept = 2 include arginine/homoarginine (C_6_H_14_N_4_O_2_/C_7_H_16_N_4_O_2_) and stearic/methyl-stearic acids (C_18_H_36_O_2_/C_19_H_38_O_2_).

**Figure 4:**
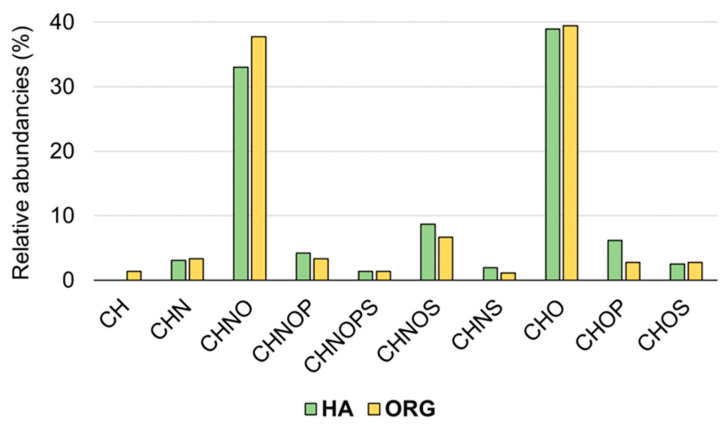
Histograms of the relative frequency of CH, CHN, CHNO, CHNOP, CHNOPS, CHNOS, CHNS, CHO, CHOP, and CHOS compounds in the hydroalcoholic phase (green) and in the organic phase (yellow).

The averaged relative frequency distribution is also displayed in the histograms of CHO, CHNO, CHN, CHNOS, CHOP, CHNOP, CHNOPS, CHNS, CH, and CHOS elemental composition (Figure 4). Both extracts are largely populated by CHO and CHNO components, with more hits in the organic portion, followed by CHNOS and CHOP species, with more entries in the hydroalcoholic extract. A small number of CH compounds are present only in the organic phase.

### 3.3. GC-MS Analysis

GC-MS methodologies allowed us to determine volatile compounds, alkanes, and fatty acids, and these are listed in Table 2, Table 3 and Table 4.

The SPME approach for the GC-MS analysis was a robust and effective method for detecting volatile metabolites in the untreated matrix, whereas the analysis of hexane extract allowed us to identify a class of molecules peculiar to insects, namely methyl-branched hydrocarbons (MBCHs).

The GC-MS technique allowed us to identify six fatty acids: linoleic acid (38.1%) and palmitic acid (27.8%) were the most abundant, followed by oleic acid (21.0%), stearic acid (10.4%), and linolenic acid (2.5%), and, with a much lower percentage value, pentanoic acid (0.2%).

## 4. Discussion

The metabolite profile obtained using the proposed analytical protocol is described and discussed for this class of compounds, combining the results obtained using the different methodologies and carrying out, when possible, a comparison with reported literature data.

*Amino acids and derivatives:* Among the nineteen amino acids identified and quantified by means of NMR, alanine and proline were shown to be the main amino acids, whereas methionine was present in the lowest concentration. All the nine essential amino acids were present, confirming the important nutritional value of edible crickets. Moreover, it is noteworthy that the NMR amino acid quantification here refers to the free amino acids naturally present in the matrix, without any protein hydrolysis. In contrast, previous studies carried out on *A. domesticus* powder [8,9] have reported the amino acid content obtained after protein hydrolysis, making a direct data comparison not possible. FT-ICR MS allowed us to also detect arginine and to reveal the presence of dipeptides (cysteinyl-methionine, methionyl-methionine, methionyl-tyrosine) and tripeptides (valyl-arginyl-tyrosine, methionyl-leucyl-phenylalanine, arginyl-prolyl-proline).

*Organic acids:* Acetate, formate, fumarate, lactate, and succinate were identified and quantified by means of NMR spectroscopy, with formate being identified in house crickets for the first time. From a quantitative point of view, lactate was the most abundant metabolite of the series, whereas fumarate was measured in the lowest concentration (365 times lower than lactate). Comparing these data with the results previously obtained for *A. domesticus* powder [12] using GC-MS analysis, it is possible to observe both similarities and differences. In particular, succinate and lactate were present in the same concentration range in both studies, whereas fumarate and acetate were shown to have different behavior. In the case reported here, fumarate was the organic acid present at the lowest acid concentration (1.87 mg/100 g), whereas in the cited paper, fumarate has been reported as the more representative acid, with a concentration 685 times higher than the one reported here, with acetate being present at the lowest concentration. Moreover, in the *A. domesticus* powder investigated here, formate was also detected, whereas Beldean at al. reported the presence of ascorbate. FT-ICR MS analysis allowed the detection of ethylmalate, a derivate of succinic acid, and sebacic acid in the hydroalcoholic extract, whereas gluconic acid was revealed in both extracts.

*Fatty acids:* Lipids in household crickets are found in the form of triglycerides stored in adipocytes, which, along with glycogen, are a relevant energy reserve and represent a largely studied class of molecules in this matrix. The GC-MS analysis of the derivatized sample of cricket powder allowed us to determine the fatty acid content, with linoleic acid and palmitic as the most abundant, followed by oleic, stearic, linolenic, and pentanoic acids. Our results are in accordance with the literature data [8,11].

The same fatty acids were also detected by means of FT-ICR MS analysis. Moreover, considering the higher sensitivity of this technique, further putative formulas of fatty acids were identified, namely lauric, myristic, and palmitoleic. Several classes of fatty acid derivatives, namely amide (palmitamide, oleamide, linoleamide, stearamide) and ester (palmitoylcarnitine and stearoylcarnitine) derivatives, were also revealed, together with some hydroxy fatty acids (hydroxy-linoleic and hydroxy-linolenic acids), well known for their anti-inflammatory, antioxidant, and anti-diabetic properties [24].

**Figure 5:**
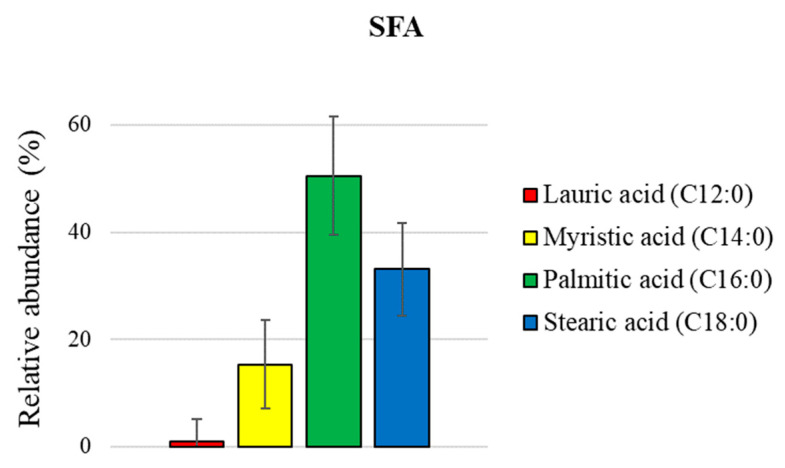
Histograms of relative distribution (mean value of the three analyzed batches ± SD) of specific classes of saturated fatty acids (SFA) obtained through ESI (−) MS analyses of organic *Acheta domesticus* powder extracts.

**Figure 6:**
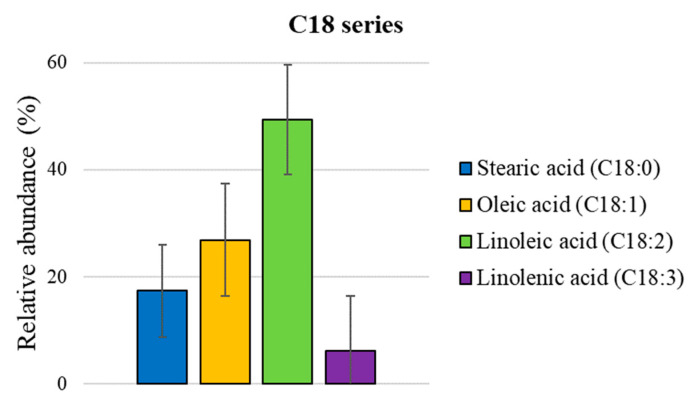
Histograms of relative distribution (mean value of the three analyzed batches ± SD) of specific classes of C18 series fatty acids obtained through ESI (−) MS analyses of organic *Acheta domesticus* powder extracts.

**Figure 7:**
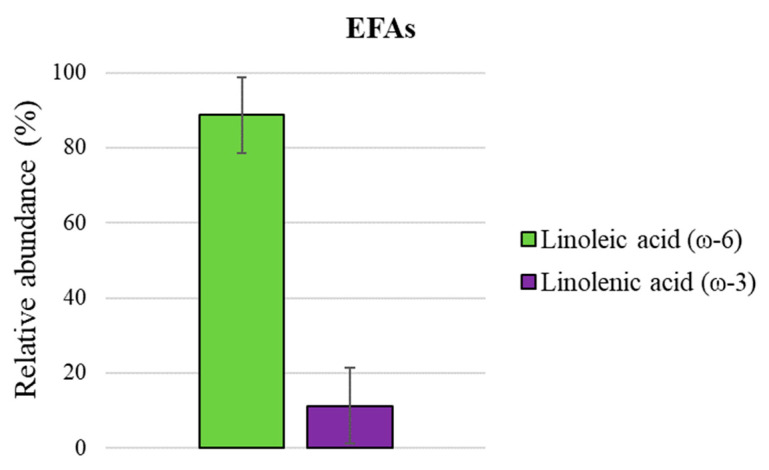
Histogram of relative distribution (mean value of the three analyzed batches ± SD) of essential fatty acids (EFAs) obtained through ESI (−) MS analyses of organic *Acheta domesticus* powder extracts: linoleic (ω-6) and linolenic acids(ω-3).

Due to a possible ion-suppression effect and variations in the signal response, ESI MS is not able to offer an accurate quantitative analysis. However, a quantification of isomeric groups of wax esters [25] and fatty acids [16] has recently been reported, since their relative ionization response depends only on lipid family and saturation degree and is not affected by carbon chain length. A similar approach was employed here, as shown in Figure 5, wherein the lipids’ molecular formulas were extrapolated from the mass list obtained through ESI (−) MS analyses of the organic extract. Then, they were summarized in two main families based on their saturation degree and carbon chain length to obtain their relative extent. Notably, in the C18 series (18:0–18:3) shown in Figure 6, linoleic acid (18:2) is the most abundant species (ca. 50%), followed by oleic (18:1) and stearic (18:0) acids, ca. 27% and 17%, respectively, whereas linolenic (18:3) acid amounts to ca. 6%. Figure 7 displays the relative abundance of EFAs (essential fatty acids), with linoleic acid (ω-6) found to be ca. ninefold more abundant than linolenic (ω-3) acid.

*Terpenes:* It has been demonstrated that insects can be a source of terpenes through both specific biosynthetic pathways that occur in insects and the accumulation of these molecules from plants [26]. In a previous study, [12] two terpenes, *p*-cymene and *β*-myrcene, were identified in *A. domesticus* powder by means of an in-tube extraction technique (ITEX) coupled with GC-MS. Here, a further characterization of house crickets’ terpene profile was carried out by means of SPME-GC-MS. Specifically, together with the previously identified *β*-myrcene and *p*-cymene, *γ*-terpinene, α-thujene, *β*-thujene, and 4-carene were detected for the first time. Among them, *γ*-terpinene (16.8%) and *p*-cymene (17.2%) were the most abundant terpenes, whereas the other metabolites were present with percentage mean values ranging from 0.7% to 3.5%. Since terpenes are a class of compounds characterized by several biological activities, it will be interesting to correlate the presence of these molecules with the potential influence of cricket extracts on human health.

*Alkanes*: The GC-MS analysis of hexane extracts allowed us to identify lipophilic compounds present in spray-dried *A. domesticus* powder, namely hexadecenoic acid and alkanes. It is noteworthy that a previous GC-MS study on *A. domesticus* PLE (pressurized liquid extraction) extract already detected a series of lipophilic compounds indicated to be “alkane”, without identifying their precise structure [10].

Here, for the first time, the elucidation of the alkane compounds present in house crickets was carried out. Specifically, thirteen alkanes were identified, namely octane, decane, and eleven methyl-branched hydrocarbons (MBCHs). MBCHs are a particular class of compounds naturally produced by insects for their inter- and intraspecific communication [27]. Among them, methylcyclopentane was the main compound present in a percentage (62.9%) at least 100 times higher than the other detected molecules. The relevant presence of methylcyclopentane has also been reported in other insects, namely Azteca ants and *A. dichotoma* [28,29]. The presence of this compound in a new food matrix such as in crickets or other edible insects will be the object of interesting research regarding the potential biological activity of these molecules, since the reported presence of this chemical class in other food sources is limited to a few studies regarding the cuticular composition of crustacea [30].

*Other metabolites*: NMR analysis allowed us to identify and quantify other metabolites belonging to different chemical classes. Among them, glycerol was the most abundant compound in the hydroalcoholic extract, with a concentration of more than 1 g/100 g of the sample. The presence of glycerol in *A. domesticus* has been previously demonstrated [31], and it represents a common metabolite of insects since this molecule has been shown to have an important role as both a cryoprotective and energy source agent. Choline was also identified by means of NMR spectroscopy and quantified at a concentration of ca. 153 mg/100 g. In addition, several vitamins were identified through FT-ICR MS analysis, including calcitriol, vitamin D3, and tocopherol. Moreover, as indicated in the van Krevelen diagrams reported in Figure 3, some hits are revealed in the area of carbohydrates. According to the putative annotations presented in Table S1, these include a disaccharide (C_12_H_22_O_11_), arabinopyranobiose (C_10_H_18_O_9_), and gluconic acid (C_6_H_12_O_7_). As expected, only a very limited number of compounds populate the CHO component, mainly corresponding to lipid and terpenoid species.

The multimethodological approach described here allowed us to obtain a rich chemical profiling of the novel food *A. domesticus*, including the identification of metabolites that had never been detected in this matrix before. These first results could represent the beginning of a new research field based on the application of advanced techniques for the characterization of edible insects and, more generally, other innovative food matrices that need to be characterized and investigated in depth. However, it is noteworthy that several issues related to edible insect safety must be clarified and studied in depth, namely the presence of aerobic bacteria, other microbial pathogens, antinutritional molecules, allergens, and heavy metals [32]. Moreover, the investigation of several practices in cricket feeding, farming, and processing could be useful for a better understanding of their influence on the insect chemical profile and, thus, its nutritional properties.

## 5. Conclusions

In this study, a complete metabolite characterization of spray-dried *A. domesticus* powder was carried out. The application of several analysis methodologies, namely NMR, FT-ICR MS, and GC-MS, allowed us to define a very rich chemical profile of this novel food. The obtained results confirmed that edible insects represent a very precious source in the perspective of having an innovative food source with a low environmental impact. The results obtained here confirmed the previously demonstrated presence of compounds important from a nutritional point of view, such as essential amino acids, organic acids, and polyunsaturated fatty acids. Moreover, two further classes of compounds, namely terpenes and methyl-branched hydrocarbons, were identified in the analyzed powder. The study of the biological activity of cricket extracts, as well as bioaccessibility and bioavailability studies, could open up new fields regarding their potential effect on human health and, thus, their use in producing new formulations.

## Figures and Tables

**Table 1:** Metabolites identified in the 600.17 MHz ^1^H-NMR spectra of spray-dried *A. domesticus* Bligh–Dyer hydroalcoholic extracts dissolved in 100 mM phosphate buffer/D_2_O containing TSP 0.4 mM. Quantitative results of the three analyzed batches are expressed as mg/100 g of sample ±SD.

Metabolite	Assignment	^1^H (ppm)	Multiplicity [*J*(Hz)]	^13^C (ppm)	mg/100 g of Sample
**Amino acids and derivatives**					
Alanine	COO^-^			176.8	931.43 ± 18.06
	α-CH	3.80	q [7.3]	51.5	
	β-CH_3_	1.49 *	d [7.3]	17.2	
					
Aspartate	α-CH	3.92		52.4	78.00 ± 2.69
	β, β’-CH_2_	2.70; 2.82 *	dd [17.4; 3.8]	37.7	
					
Betaine	N(CH_3_)_3_^+^	3.27 *	s	54.6	100.16 ± 1.44
	α-CH_2_			67.7	
					
Citrulline	α-CH	3.14 *	m		397.96 ± 8.38
	β-CH_2_	1.89			
	γ-CH_2_	1.60			
					
Glycine	COO^-^			175.0	631.41 ± 35.29
	α-CH_2_	3.57 *	s	42.6	
					
Glutamate	α-CH	3.78		55.6	464.30 ± 19.24
	β, β’-CH_2_	2.07; 2.14		28.0	
	γ-CH_2_	2.36 *	m	34.8	
	δ-COO^-^			182.6	
					
Glutamine	α-CH	3.81			265.74 ± 11.73
	β, β’-CH_2_	2.15			
	γ-CH_2_	2.46 *	m	31.8	
					
Histidine	CH-3, ring	8.04 *	s		120.36 ± 6.88
	CH-5, ring	7.15	s		
					
Isoleucine	α-CH	3.69		60.7	189.50 ± 9.24
	β-CH	1.99		37.0	
	γ-CH_3_	1.02 *	d [7.1]	15.8	
	γ’-CH	1.29		25.6	
	δ-CH_3_	0.94	t [7.4]	12.3	
					
Leucine	α-CH	3.74		54.4	417.98 ± 3.21
	β-CH_2_	1.72		40.8	
	δ, δ’-CH_3_	0.96; 097 *	d [6.2]	23.0	
					
Lysine	α-CH	3.74		54.5	351.05 ± 9.41
	β-CH_2_	1.91		30.9	
	γ -CH_2_	1.48		21.2	
	δ-CH_2_	1.75		27.5	
	ε-CH_2_	3.02 *	t [7.3]	40.2	
					
Methionine	α-CH	3.74		55.0	23.69 ± 1.58
	β-CH_2_	2.25	m	30.5	
	γ-CH_2_	2.65 *	t [7.4]	30.0	
	S-CH_3_	2.16	s	14.9	
					
Phenylalanine	CH-2,6 ring	7.32	m	130.5	167.43 ± 5.08
	CH-3,5 ring	7.42 *	m	130.1	
	CH-4 ring	7.37	m	128.7	
					
Proline	α-CH	4.15			977.76 ± 17.27
	β, β’-CH_2_	2.06; 2.35	m		
	γ-CH_2_	2.01 *	m	25.3	
	δ, δ’-CH_2_	3.35; 3.43	m		
					
Taurine	S-CH_2_	3.28	t [6.5]	48.4	259.83 ± 13.73
	N-CH_2_	3.44 *	t [6.5]	36.3	
					
Threonine	α-CH	3.60		61.4	88.89 ± 6.86
	β-CH	4.27		67.1	
	γ-CH_3_	1.34 *	d [6.6]	21.2	
					
Tryptophan	CH-4, ring	7.72	d [8.1]		73.10 ± 2.54
	CH-5, ring	7.20			
	CH-6, ring	7.27			
	CH-7, ring	7.51 *	d [8.1]		
					
Tyrosine	CH-2,6 ring	6.89 *	d [8.6]	116.9	537.32 ± 4.82
	CH-3,5 ring	7.20	d [8.6]	129.5	
	C-4 ring			155.5	
					
Valine	α-CH	3.63		61.6	390.18 ± 17.35
	β-CH	2.30		30.3	
	γ-CH_3_	0.99	d [7.06]	18.0	
	γ’-CH_3_	1.05	d [7.06]	19.1	
**Organic acids**					
Acetate	COO^-^			182.5	359.64 ± 16.25
	CH_3_	1.92 *	s	24.4	
					
Formate	HCOO^-^	8.47 *	s		22.80 ± 0.42
					
Fumarate	α, β-CH=CH	6.53 *	s		1.87 ± 0.09
					
Lactate	COO^-^			183.5	730.94 ± 11.40
	α-CH	4.12		69.6	
	β-CH_3_	1.33 *	d [6.6]	22.6	
					
Succinate	COO^-^			183.7	411.96 ± 8.59
	α, β-CH_2_	2.41 *	s	35.1	
**Other metabolites**					
Choline	N(CH_3_)_3_^+^	3.21 *	s	55.2	153.21 ± 1.54
	α-CH_2_			68.9	
					
Glycerol	CH-1,3	3.56 *	dd [11.7; 6.5]	63.6	1175.31 ± 23.08
	CH_2_-2	3.79	m	55.4	
	CH-1′,3′	3.66	dd [11.7; 4.3]	63.6	

Asterisks (*) indicate signals selected for integration.

**Table 2:** Volatile profile (percentage mean value of the three batches ±SD) of *A. domesticus* powder.

N°	Component ^1^	LRI ^2^	LRI ^3^	(%)
1	*α*-thujene	820	823	0.9 ± 0.02
2	*β*-thujene	962	968	0.7 ± 0.03
3	*β*-myrcene	985	983	3.5 ± 0.08
4	4-carene	1008	1001	1.5 ± 0.02
5	*p*-cymene	1015	1013	17.2 ± 0.02
6	1,2-dipropenyl-cyclobutane	1021	*	1.5 ± 0.02
7	*γ*-terpinene	1051	1054	16.8 ± 0.03
8	6-ethyl-2-methyl-decane	1351	*	2.5 ± 0.03
9	linalyl butyrate	1406	1402	0.8 ± 0.02
10	hexadecanoic acid	1968	1973	54.6 ± 0.02
	SUM			100.0

^1^ The components are reported according to their elution order on a polar column; ^2^ linear retention indices measured on a polar column; ^3^ linear retention indices from the literature; * LRI not available.

**Table 3:** Chemical composition (percentage mean value of the three batches ±SD) of *A. domesticus* hexane extract.

N°	Component ^1^	LRI ^2^	LRI ^3^	(%)
1	methylcyclopentane	635	629	62.9 ± 0.04
2	4-methyl-heptane	771	768	0.2 ± 0.02
3	octane	810	*	0.1 ± 0.02
4	2,4-dimethyl-1-heptane	822	821	0.2 ± 0.02
5	2,3,4-trimethyl-hexane	854	850	0.1 ± 0.02
6	3,3-dimethyl-octane	930	935	0.2 ± 0.01
7	2,3,6,7-tetramethyl-octane	928	935.5	0.3 ± 0.02
8	decane	1010	*	0.3 ± 0.01
9	4,7-dimethyl-undecane	1121	*	0.2 ± 0.02
10	4-methyl-undecane	1167	1160	0.6 ± 0.02
11	2,5-dimethyl-benzaldehyde	1215	1208	0.2 ± 0.02
12	4,6-dimethyl-dodecane	1335	1325*	0.7 ± 0.02
13	2,4-di-tert-butylphenol	1529	1521	0.7 ± 0.02
14	hexadecanoic acid	1980	1973	32.8 ± 0.04
	SUM			99.5

^1^ The components are reported according to their elution order on a polar column; ^2^ linear retention indices measured on a polar column; ^3^ linear retention indices from the literature; * LRI not available.

**Table 4:** Fatty acid composition (percentage mean value of the three batches ±SD) of *A. domesticus*.

N°	Component ^1^	LRI ^2^	LRI ^3^	(%)
1	pentanoic acid	1758	1762	0.2 ± 0.03
2	palmitic acid	2941	2946	27.8 ± 0.05
3	stearic acid	3183	3181	10.4 ± 0.03
4	oleic acid	3190	3184	21.0 ± 0.07
5	linoleic acid	3217	*	38.1 ± 0.06
6	linolenic acid	3289	3292	2.5 ± 0.02
	SUM			100.0

^1^ The components are reported according to their elution order on a polar column; ^2^ linear retention indices measured on a polar column; ^3^ linear retention indices from the literature; * LRI not available.

## Data Availability

All data reported in this study are available within the article.

## Supplementary Materials

The following supporting information can be downloaded at: https://www.mdpi.com/article/10.3390/foods12122331/s1, Table S1: Untargeted metabolic profiling of hydroalcoholic and organic extracts of *A. domesticus* powder.

## References

[1] Mariutti L.R.B., Rebelo K.S., Bisconsin-Junior A., de Morais J.S., Magnani M., Maldonade I.R., Madeira N.R., Tiengo A., Maróstica M.R., Cazarin C.B.B. (2021). The use of alternative food sources to improve health and guarantee access and food intake. Food Res. Int..

[2] United Nations Sustainable Development GOALS. https://www.un.org/sustainabledevelopment/development-agenda/.

[3] Baiano A. (2020). Edible insects: An overview on nutritional characteristics, safety, farming, production technologies, regulatory framework, and socio-economic and ethical implications. Trends Food Sci. Technol..

[4] van Huis A. (2020). Insects as food and feed, a new emerging agricultural sector: A review. J. Insects Food Feed.

[5] Kouřimská L., Adámková A. (2016). Nutritional and sensory quality of edible insects. NFS J..

[6] FAO (2013). Edible Insects—Future Prospects for Food and Feed Security.

[7] Union E. EUR-Lex. https://eur-lex.europa.eu/legal-content/EN/TXT/?uri=CELEX:32022R0188.

[8] Khatun H., Claes J., Smets R., De Winne A., Akhtaruzzaman M., Van Der Borght M. (2021). Characterization of freeze-dried, oven-dried and blanched house crickets (*Acheta domesticus*) and Jamaican field crickets (*Gryllus assimilis*) by means of their physicochemical properties and volatile compounds. Eur. Food Res. Technol..

[9] Udomsil N., Imsoonthornruksa S., Gosalawit C., Ketudat-Cairns M. (2019). Nutritional Values and Functional Properties of House Cricket (*Acheta domesticus*) and Field Cricket (*Gryllus bimaculatus*). Food Sci. Technol. Res..

[10] Navarro del Hierro J., Gutiérrez-Docio A., Otero P., Reglero G., Martin D. (2020). Characterization, antioxidant activity, and inhibitory effect on pancreatic lipase of extracts from the edible insects *Acheta domesticus* and *Tenebrio molitor*. Food Chem..

[11] Grapes M., Whiting P., Dinan L. (1989). Fatty acid and lipid analysis of the house cricket, *Acheta domesticus*. Insect Biochem..

[12] Beldean B.V., Chiș M.S., Alexa E., Pop C., Păucean A., Man S., Igual M., Haydee K.M., Dalma K.E., Stănilă S. (2022). The Impact of Insect Flour on Sourdough Fermentation-Fatty Acids, Amino-Acids, Minerals and Volatile Profile. Insects.

[13] Nino M.C., Reddivari L., Ferruzzi M.G., Liceaga A.M. (2021). Targeted phenolic characterization and antioxidant bioactivity of extracts from edible *Acheta domesticus*. Foods.

[14] Spano M., Di Matteo G., Ingallina C., Botta B., Quaglio D., Ghirga F., Balducci S., Cammarone S., Campiglia E., Giusti A.M. (2021). A multimethodological characterization of cannabis sativa l. Inflorescences from seven dioecious cultivars grown in Italy: The effect of different harvesting stages. Molecules.

[15] Ingallina C., Spano M., Sobolev A.P., Esposito C., Santarcangelo C., Baldi A., Daglia M., Mannina L. (2020). Characterization of Local Products for Their Industrial Use: The Case of Italian Potato Cultivars Analyzed by Untargeted and Targeted Methodologies. Foods.

[16] Spano M., Maccelli A., Di Matteo G., Ingallina C., Biava M., Crestoni M.E., Bardaud J.X., Giusti A.M., Mariano A., D’abusco A.S. (2021). Metabolomic profiling of fresh goji (*Lycium barbarum* L.) berries from two cultivars grown in central Italy: A multi-methodological approach. Molecules.

[17] Ruggeri M., Bianchi E., Vigani B., Sánchez-Espejo R., Spano M., Totaro Fila C., Mannina L., Viseras C., Rossi S., Sandri G. (2023). Nutritional and Functional Properties of Novel Italian Spray-Dried Cricket Powder. Antioxidants.

[18] Dossey A.T., Tatum J.T., McGill W.L. (2016). Modern Insect-Based Food Industry: Current Status, Insect Processing Technology, and Recommendations Moving Forward. Insects as Sustainable Food Ingredients: Production, Processing and Food Applications.

[19] Di Matteo G., Spano M., Esposito C., Santarcangelo C., Baldi A., Daglia M., Mannina L., Ingallina C., Sobolev A.P. (2021). Nmr characterization of ten apple cultivars from the piedmont region. Foods.

[20] Maia M., Ferreira A.E.N., Nascimento R., Monteiro F., Traquete F., Marques A.P., Cunha J., Eiras-Dias J.E., Cordeiro C., Figueiredo A. (2020). Integrating metabolomics and targeted gene expression to uncover potential biomarkers of fungal/oomycetes-associated disease susceptibility in grapevine. Sci. Rep..

[21] Farinon B., Costantini L., Molinari R., Di Matteo G., Garzoli S., Ferri S., Ceccantoni B., Mannina L., Merendino N. (2022). Effect of malting on nutritional and antioxidant properties of the seeds of two industrial hemp (*Cannabis sativa* L.) cultivars. Food Chem..

[22] Wägele B., Witting M., Schmitt-Kopplin P., Suhre K. (2012). Masstrix reloaded: Combined analysis and visualization of transcriptome and metabolome data. PLoS ONE.

[23] Kim S., Kramer R.W., Hatcher P.G. (2003). Graphical Method for Analysis of Ultrahigh-Resolution Broadband Mass Spectra of Natural Organic Matter, the Van Krevelen Diagram. Anal. Chem..

[24] Gowda S.G.B., Fuda H., Tsukui T., Chiba H., Hui S.P. (2020). Discovery of eicosapentaenoic acid esters of hydroxy fatty acids as potent nrf2 activators. Antioxidants.

[25] Chen J., Green K.B., Nichols K.K. (2013). Quantitative profiling of major neutral lipid classes in human meibum by direct infusion electrospray ionization mass spectrometry. Investig. Ophthalmol. Vis. Sci..

[26] Beran F., Köllner T.G., Gershenzon J., Tholl D. (2019). Chemical convergence between plants and insects: Biosynthetic origins and functions of common secondary metabolites. New Phytol..

[27] Bello J.E., McElfresh J.S., Millar J.G. (2015). Isolation and determination of absolute configurations of insect-produced methyl-branched hydrocarbons. Proc. Natl. Acad. Sci. USA.

[28] Lee M.H., Kim T.K., Cha J.Y., Yong H.I., Kang M.C., Jang H.W., Choi Y.S. (2022). Physicochemical characteristics and aroma patterns of oils prepared from edible insects. LWT.

[29] Jardine K.J., de O. Piva L.R., Rodrigues T.B., Spanner G.C., Rodrigues J.R., Menezes V.S., Sampaio I., Oliveira D.C., Gimenez B.O., Higuchi N. (2020). Volatiles defenses of Amazon Azteca ants (repellent ants). bioRxiv.

[30] Hamilton R.J., Raie M.Y., Weatherston I., Brooks C.J., Borthwick J.H. (1975). Crustacean surface waxes. Part I. The hydrocarbons from the surface of *Ligia oceanica*. J. Chem. Soc. Perkin Trans..

[31] Izumiyama S., Suzuki K., Miya K. (1983). Glycerol in the Eggs of the Two-Spotted Cricket, *Gryllus bimaculatus* de Geer. Appl. Entomol. Zool..

[32] Fernandez-Cassi X., Supeanu A., Jansson A., Boqvist S., Vagsholm I. (2018). Novel foods: A risk profile for the house cricket (*Acheta domesticus*). EFSA J..

