# Supplementary material for: A Multimethodological Approach for the Chemical Characterization of Edible Insects: The Case Study of Acheta domesticus
Source: Foods. 2023 Jun 9;12(12):2331. doi: 10.3390/foods12122331 (PMC10297444; doi:10.3390/foods12122331)
Supplement: Supplementary file 1 [file foods-12-02331-s001.zip › foods-2422783-supplementary.pdf]

Supplementary Materials

A multimethodological approach for the chemical characterization of edible insects: the case study of *Acheta domesticus*

Mattia Spano [1,2], Giacomo Di Matteo [1,2], Carlos Alberto Fernandez Retamozo [1,2], Alba Lasalvia [1], Marco Ruggeri [3], Giuseppina Sandri [3], Carlos Cordeiro [4], Marta Sousa Silva [4], Carlotta Totaro Fila [5], Stefania Garzoli [1], Maria Elisa Crestoni[1], Luisa Mannina [1,2*]

*Corresponding author: luisa.mannina@uniroma1.it

Table S1. Untargeted metabolic profiling of hydroalcoholic and organic extracts of *A. domesticus* powder.

#	Putative Annotation[a]	Molecular Formula	Theo. m/z[b]	HA1[c]	HA2[c]	HA3[c]	Org1[c]	Org2[c]	Org3[c]
1	Phosphocysteamine	C2H8NO3PS	158.00277	2.15 * 10-6	2.33 * 10-6	2.16 * 10-3			
2	Sodium phosphate	Na3O4P	164.92993	3.65 * 10-6	2.98 * 10-5	3.57 * 10-4			
3	cis-4-Decenoic acid	C10H18O2	171.13803				1.60 * 10-5	2.16 * 10-6	1.90 * 10-5
4	Sulfinoalanine	C3H6NO4S	174.99097	8.70 * 10-5					
5	Arginine	C6H14N4O2	175.11888	3.30 * 10-6	3.43 * 10-6	3.13 * 10-6			
6	Methylenediphosphonic acid	CH6O6P2	176.97062	1.92 * 10-6	2.24 * 10-4	2.26 * 10-5			1.76 * 10-4
7	Undecynoic acid	C11H18O2	183.13796					1.76 * 10-2	1.81 * 10-2
8	Ethylmalate	C6H10O5	185.04204	2.28 * 10-5	2.23 * 10-2	2.18 * 10-2			
9	Methyl-3-decenoic acid	C11H20O2	185.15355				3.28 * 10-6	4.68 * 10-4	3.76 * 10-4
10	Alanylproline	C8H14N2O3	187.10767	2.35 * 10-5	2.26 * 10-6	2.49 * 10-6			
11	Tridecatrienal	C13H20O	193.15869					1.77 * 10-2	1.57 * 10-2
12	Shikimic acid	C7H10O5	197.04204	2.73 * 10-4	3.40 * 10-1	2.67 * 10-2			
13	Dihydro-octenyl-furanone	C12H20O2	197.15357				4.18 * 10-6	4.49 * 10-6	4.55 * 10-6
14	Linderic acid	C12H22O2	199.16926				3.12 * 10-2	3.91 * 10-2	4.90 * 10-1
15	Lauric acid	C12H24O2	201.18491			1.76 * 10-2	2.73 * 10-2	2.70 * 10-1	2.73 * 10-2
16	Amino-undecanoic acid	C11H23NO2	202.18016	2.23 * 10-5			2.30 * 10-7	2.26 * 10-7	2.24 * 10-7
17	6-Acetyl-2,2-dimethyl-2H-1-benzopyran	C13H14O2	203.10693		1.78 * 10-6	1.72 * 10-5			
18	Anofinic acid	C12H12O3	205.08589				2.48 * 10-5	1.98 * 10-6	1.98 * 10-6
19	Tryptophan	C11H12N2O2	205.09712	2.68 * 10-5	2.72 * 10-4	2.95 * 10-5			
20	Tetradecatrienal	C14H22O	207.17434					1.86 * 10-2	2.45 * 10-2

**21**	Heptyloxyphenol	C13H20O2	209.15361	4.98 * 10-5	5.33 * 10-2	5.62 * 10-2	9.87 * 10-2	9.54 * 10-2	1.00E+00
**22**	Homoarginine	C7H16N4O2	211.11655	2.35 * 10-3	2.30 * 10-1	2.27 * 10-2			
**23**	Linalyl propionate	C13H22O2	211.16926				5.80 * 10-4	6.70 * 10-6	7.26 * 10-6
**24**		C12H21NO2	212.16451				1.97 * 10-6	1.89 * 10-6	2.40 * 10-6
**25**	Methyl-2-dodecenoic acid	C13H24O2	213.18491	1.91 * 10-5			2.73 * 10-2	3.88 * 10-2	3.83 * 10-2
**26**		C12H23NO2	214.18015					1.63 * 10-6	1.76 * 10-6
**27**	Trimethylolmelamine	C6H12N6O3	217.10463	8.46 * 10-5	7.68 * 10-5	7.78 * 10-5	2.48 * 10-6	2.24 * 10-6	2.83 * 10-6
**28**	Gluconic acid	C6H12O7	219.04752	3.62 * 10-5	2.73 * 10-2	3.22 * 10-2			
**29**	Succimer	C4H6O4S2	220.93391	1.65 * 10-5					
**30**	Acetyl-2-hydroxyphenyl-3-methyl-1-butanone	C13H16O3	221.11721				1.65 * 10-6	1.94 * 10-5	2.88 * 10-5
**31**		C16H28	221.22637					1.89 * 10-6	1.77 * 10-6
**32**	Decenedioic acid	C10H16O4	223.09408	6.90 * 10-4	7.35 * 10-2	6.85 * 10-2	2.95 * 10-2	2.96 * 10-2	2.58 * 10-2
**33**	Annuionone B	C13H18O3	223.13287					1.63 * 10-6	1.64 * 10-4
**34**	Serine O-sulfate	C3H7NO6S	223.96257	7.24 * 10-5					
**35**	Phosphoglycerate	C3H7O7P	224.95610	5.87 * 10-5					
**36**	Sebacic acid	C10H18O4	225.10973	9.69 * 10-5	9.65 * 10-2	9.93 * 10-2	3.72 * 10-2	3.68 * 10-2	3.99 * 10-2

#	Putative Annotation[a]	Molecular Formula	Theo. m/z[b]	HA1[c]	HA2[c]	HA3[c]	Org1[c]	Org2[c]	Org3[c]
**37**	Tetradecadienoic acid	C14H24O2	225.18491				5.61 * 10-2	6.96 * 10-2	7.70 * 10-1
**38**		C8H2O4S2	226.94679	4.00 * 10-6	2.98 * 10-6	2.34 * 10-6			
**39**	Diethyl -2-methyl-3-hydroxysuccinate	C9H16O5	227.08899	1.91 * 10-4	1.86 * 10-2	1.92 * 10-2			
**40**	Myristoleic acid	C14H26O2	227.20056	1.97 * 10-5	1.99 * 10-2		4.18 * 10-2	4.11 * 10-2	3.85 * 10-2
**41**		C13H25NO2	228.19579	4.66 * 10-6	4.97 * 10-6	5.97 * 10-6	1.93 * 10-7	2.13 * 10-7	2.31 * 10-7
**42**	Dodecenedioic acid	C12H20O4	229.14344				1.74 * 10-2	1.55 * 10-2	2.50 * 10-1
**43**	Isoleucylproline	C11H20N2O3	229.15466	2.12 * 10-5	2.59 * 10-6	2.43 * 10-4			
**44**	Myristic acid	C14H28O2	229.21621	6.14 * 10-4	6.36 * 10-2	6.63 * 10-2	1.32 * 10-2	1.27 * 10-2	1.32 * 10-2
**45**	Xestoaminol C	C14H31NO	230.24784	5.82 * 10-6	5.80 * 10-6	5.83 * 10-6	1.84 * 10-5	2.22 * 10-5	2.11 * 10-4
**46**	Dimethyl-dioxo-dodecatrienal	C14H18O3	235.13287				2.14 * 10-2	1.97 * 10-2	2.24 * 10-2
**47**	S-aminomethyldihydrolipoamide	C9H20N2OS2	237.10898	4.29 * 10-5	4.48 * 10-2	4.79 * 10-2		1.98 * 10-2	1.54 * 10-2
**48**	Heptyloxybenzoic acid	C14H20O3	237.14852				1.86 * 10-2	1.57 * 10-2	1.84 * 10-2
**49**	Methyl lauric acid	C13H26O2	237.18250		2.50 * 10-1	2.12 * 10-2			
**50**	Hexadecadienal	C16H28O	237.22129		1.83 * 10-2		4.78 * 10-2	4.29 * 10-2	4.18 * 10-2
**51**	Carbamoyl-2-hydroxyethyl guanine	C8H10N6O3	239.08900	8.56 * 10-6	7.99 * 10-5	8.78 * 10-5	3.82 * 10-6	3.39 * 10-5	3.32 * 10-6
**52**	Methoxychalcone	C16H14O2	239.10666	1.84 * 10-5	1.92 * 10-2	2.70 * 10-1	3.96 * 10-2	3.78 * 10-2	3.62 * 10-2
**53**	Geranyl 3-methylbutanoate	C15H26O2	239.20057				2.19 * 10-6	2.19 * 10-5	2.28 * 10-4
**54**	3-Methylcyclopentadecanone	C16H30O	239.23694	2.01 * 10-6	2.13 * 10-6	2.13 * 10-6	5.30 * 10-6	5.67 * 10-6	5.73 * 10-6
**55**	Dioscoretine	C13H23NO3	242.17506				1.89 * 10-6	1.58 * 10-5	1.96 * 10-6
**56**		C14H27NO2	242.21145			1.88 * 10-6	5.02 * 10-6	5.64 * 10-6	5.25 * 10-6
**57**	Diethylene glycol dimethacrylate	C12H18O5	243.12271				1.88 * 10-6	1.76 * 10-6	1.79 * 10-5
**58**		C18H26	243.21074				3.42 * 10-6	3.51 * 10-6	3.78 * 10-6
**59**	Methyl myristic acid	C15H30O2	243.23186	5.75 * 10-5	6.83 * 10-2	6.95 * 10-2	1.70 * 10-1	1.20 * 10-1	9.66 * 10-2
**60**		C5HN5OS3	243.94165	4.88 *	4.55 * 10-	3.74 *			

				10-6	6	10-6			
**61**	Undecanoylglycine	C13H25NO3	244.19072	2.51 * 10-4	2.72 * 10-2	3.00E+00	5.49 * 10-2	5.60 * 10-1	5.53 * 10-2
**62**	Dodecatetraenedioic acid	C12H14O4	245.07843	7.33 * 10-5					
**63**		C7H17O7P	245.07844	7.33 * 10-6	7.56 * 10-6	6.92 * 10-6	3.50 * 10-6	3.50 * 10-6	3.10 * 10-6
**64**	Methyl-tetradecahydrocyclopenta phenanthrene	C18H28	245.22639				2.34 * 10-6	2.75 * 10-6	2.72 * 10-7
**65**	Dodecylbenzene	C18H30	247.24204				3.76 * 10-4	4.92 * 10-4	5.75 * 10-6
**66**	Hydroxyjasmonic acid	C12H18O4	249.10973	3.56 * 10-4		3.52 * 10-2	1.63 * 10-2		
**67**	Methyl dihydrojasmonate	C13H22O3	249.14612	7.98 * 10-6	8.16 * 10-3	8.17 * 10-5	3.34 * 10-6	3.15 * 10-4	3.24 * 10-5
**68**	Diaminopurine-dideoxyriboside	C10H14N6O2	251.12539	6.48 * 10-6	6.12 * 10-6	6.33 * 10-4	2.47 * 10-5	2.43 * 10-6	2.84 * 10-6
**69**	Hexadecatetraenoic acid	C16H26O2	251.20056				1.90 * 10-1	1.58 * 10-2	
**70**	Decanoylglycine	C12H23NO3	252.15701	5.20 * 10-5	5.52 * 10-2	4.62 * 10-2	2.63 * 10-2	2.89 * 10-2	2.45 * 10-2
**71**	Cysteinyl-Methionine	C8H16N2O3S2	253.06826		1.82 * 10-5	2.18 * 10-5			
**72**	Palmitolinoleic acid	C16H28O2	253.21621			2.60 * 10-1	4.30 * 10-1	3.92 * 10-2	3.69 * 10-2
**73**	Methyl-dihydroxy-methylbutyl-4-hydroxybenzoate	C13H18O5	255.12273				2.80 * 10-5	1.73 * 10-5	

#	Putative Annotation[a]	Molecular Formula	Theo. m/z[b]	HA1[c]	HA2[c]	HA3[c]	Org1[c]	Org2[c]	Org3[c]
**74**	Heptadecanal	C17H34O	255.26825			1.86 * 10-5	2.24 * 10-5	2.27 * 10-5	2.50 * 10-5
**75**		C7H5N5S3	255.97804	3.19 * 10-6	2.30 * 10-6	1.83 * 10-6			
**76**	Palmitamide	C16H33NO	256.26349				6.80 * 10-1	6.63 * 10-2	6.14 * 10-2
**77**		C17H37N	256.29989	3.29 * 10-7	3.11 * 10-7	3.07 * 10-7	2.24 * 10-7	2.17 * 10-7	2.05 * 10-7
**78**	Methoxy-trimethoxyphenyl-2-propanol	C13H20O5	257.13836				1.67 * 10-6	1.62 * 10-6	1.57 * 10-6
**79**	Valerenic acid	C15H22O2	257.15120	6.91 * 10-5	6.41 * 10-2	6.35 * 10-2	2.69 * 10-2	2.38 * 10-2	2.29 * 10-2
**80**	Hexadecatrienal	C16H26O	257.18759	4.23 * 10-5	4.11 * 10-2	4.70 * 10-1			
**81**	Palmitic acid	C16H32O2	257.24751	3.56 * 10-5	4.29 * 10-2	4.91 * 10-2	1.94 * 10-2	2.13 * 10-2	2.21 * 10-2
**82**	Hexenoylcarnitine	C13H23NO4	258.16998	9.14 * 10-5	8.94 * 10-2	8.50 * 10-1	1.21 * 10-2	1.90 * 10-1	1.14 * 10-2
**83**	Lauroylglycine	C14H27NO3	258.20637					1.93 * 10-2	1.55 * 10-2
**84**		C16H35NO	258.27915	3.57 * 10-6	3.52 * 10-6	3.73 * 10-6			
**85**	Tetradecanedioic acid	C14H26O4	259.19039				2.28 * 10-2	1.96 * 10-2	1.86 * 10-2
**86**	Glucose 1-phosphate	C6H13O9P	261.03700				2.93 * 10-2	1.60 * 10-1	
**87**	Carboxy-methyl-propyl-furanpropanoic acid	C12H16O5	263.08899	2.73 * 10-5					
**88**		C7H19O8P	263.08900	2.73 * 10-6	2.53 * 10-6	2.58 * 10-6			
**89**	Phosphatidylglycerol	C8H23O7P	263.12538	3.21 * 10-6	2.99 * 10-6	3.48 * 10-6			
**90**	Tridecenyl acetate	C15H28O2	263.19815	2.88 * 10-5	3.48 * 10-2	3.20 * 10-1			
**91**	Dodecylphenol	C18H30O	263.23694				1.16 * 10-2	1.38 * 10-2	1.44 * 10-2
**92**	Hydroxy-1-(4-hydroxyphenyl)-3-decanone	C16H24O3	265.17982				1.91 * 10-2	1.63 * 10-2	
**93**	Octadecadienal	C18H32O	265.25259				1.79 * 10-2	2.51 * 10-2	2.99 * 10-2
**94**	Aminopurin-9-yl_purin-2-amine	C10H8N10	269.09957	3.62 * 10-6	3.69 * 10-5	3.25 * 10-5	1.77 * 10-5	1.65 * 10-6	
**95**	Kamahine C	C14H20O5	269.13838					1.73 * 10-6	1.78 * 10-4
**96**	Methyl-hexadecenoic acid	C17H32O2	269.24751		1.76 * 10-2	1.93 * 10-2	3.88 * 10-2	3.50 * 10-1	3.47 * 10-2
**97**		C8H7N5S3	269.99371	2.08 *	1.73 * 10-				

				10-6	6				
**98**	Capsiamide	C17H35NO	270.27916	2.55 * 10-5	2.74 * 10-6	2.43 * 10-5	2.87 * 10-6	2.45 * 10-6	2.59 * 10-5
**99**	Prenyl glucoside	C11H20O6	271.11522	1.12 * 10-7	1.51 * 10-6	1.99 * 10-5	5.85 * 10-5	5.56 * 10-6	4.90 * 10-6
**100**	1,5-Dihydro-7-_1-piperidinyl_-imidazo_2,1-b _quinazolin-2_3H_-one	C15H18N4O	271.15401			1.75 * 10-6		2.58 * 10-6	2.04 * 10-6
**101**	Keto palmitic acid	C16H30O3	271.22677		1.72 * 10-2	1.73 * 10-2	3.51 * 10-2	3.25 * 10-2	3.49 * 10-2
**102**	Heptadecanoic acid	C17H34O2	271.26317	6.79 * 10-6	8.14 * 10-6	8.78 * 10-6	2.97 * 10-7	3.25 * 10-7	3.40 * 10-7
**103**	Hept-3-enoylcarnitine	C14H25NO4	272.18565	2.93 * 10-4	2.98 * 10-6	3.12 * 10-4	2.81 * 10-6	2.97 * 10-6	3.59 * 10-4
**104**	Amino-hexadecanoic acid	C16H33NO2	272.25841	4.65 * 10-5	4.46 * 10-2	4.74 * 10-2	3.19 * 10-2	2.88 * 10-2	3.10 * 10-1
**105**	Phosphosulfolactate	C3H7O9PS	272.94406	4.00 * 10-5	3.60 * 10-1	2.18 * 10-2			
**106**	Ubiquinone-1	C14H18O4	273.10975	7.62 * 10-6	7.86 * 10-6	8.25 * 10-6	3.75 * 10-5	4.49 * 10-4	3.88 * 10-6
**107**	Abscisic alcohol	C15H22O3	273.14612	2.44 * 10-5					
**108**		C10H25O6P	273.14613	2.44 * 10-6	2.50 * 10-6	2.44 * 10-6			
**109**	Hydroxyhex-4-enoylcarnitine	C13H23NO5	274.16493				1.65 * 10-6	2.00 * 10-6	1.77 * 10-5
**110**		C16H35NO2	274.27409				2.37 * 10-6	2.70 * 10-6	2.88 * 10-6

#	Putative Annotation[a]	Molecular Formula	Theo. m/z[b]	HA1[c]	HA2[c]	HA3[c]	Org1[c]	Org2[c]	Org3[c]
**111**	Methyl-D-erythritol-2,4-cyclodiphosphate	C5H10O9P2	276.98671	8.64 * 10-6	6.47 * 10-5	5.32 * 10-5	2.24 * 10-5	1.88 * 10-6	2.25 * 10-5
**112**	Palmitoleic acid	C16H30O2	277.21380	1.32 * 10-5	1.52 * 10-2	1.62 * 10-2	9.31 * 10-2	8.94 * 10-2	9.12 * 10-2
**113**	Leucyl-phenylalanine	C15H22N2O3	279.17032		1.71 * 10-2		2.90 * 10-1	2.70 * 10-1	1.62 * 10-2
**114**	Sodium palmitate	C16H31NaO2	279.22828				3.27 * 10-6	3.55 * 10-6	3.55 * 10-6
**115**		C18H30O2	279.23188				1.03 * 10-8	1.14 * 10-8	1.18 * 10-8
**116**	Glycerophosphocholine	C8H20NO6P	280.09204	4.42 * 10-5	3.80 * 10-1	4.26 * 10-2			
**117**	Linoleamide	C18H33NO	280.26349				2.54 * 10-2	2.65 * 10-2	2.59 * 10-2
**118**	Methionyl-Methionine	C10H20N2O3S2	281.09958	6.93 * 10-4	5.33 * 10-6	5.19 * 10-6	3.97 * 10-5		
**119**	Linoleic acid	C18H32O2	281.24751		2.35 * 10-2	2.43 * 10-2	2.84 * 10-2	3.41 * 10-2	3.67 * 10-2
**120**	Oleamide	C18H35NO	282.27914			1.78 * 10-2	2.33 * 10-2	2.32 * 10-2	2.36 * 10-2
**121**	Arabinopyranobiose	C10H18O9	283.10275	2.38 * 10-6	2.72 * 10-5	2.89 * 10-6			5.32 * 10-5
**122**	Octyl gallate	C15H22O5	283.15402	1.64 * 10-5		1.68 * 10-6	3.12 * 10-5	3.88 * 10-5	2.78 * 10-6
**123**	Oleic acid	C18H34O2	283.26316	4.23 * 10-4	6.50 * 10-1	6.12 * 10-2	9.49 * 10-2	1.39 * 10-2	1.63 * 10-2
**124**	Stearamide	C18H37NO	284.29479				3.71 * 10-2	4.60 * 10-1	4.75 * 10-2
**125**		C19H41N	284.33119	1.98 * 10-7	1.87 * 10-7	1.92 * 10-7	5.71 * 10-6	5.75 * 10-6	6.01 * 10-6
**126**	α-dihydroartemisinin	C15H24O5	285.16967				2.01 * 10-6	1.72 * 10-6	1.98 * 10-6
**127**	Stearic acid	C18H36O2	285.27881	1.42 * 10-5	1.51 * 10-2	1.69 * 10-2	3.38 * 10-2	4.50 * 10-1	5.57 * 10-2
**128**	Octenoylcarnitine	C15H27NO4	286.20128	4.69 * 10-4	4.60 * 10-1	4.10 * 10-1	5.39 * 10-2	4.89 * 10-2	4.67 * 10-2
**129**	Phlorin	C12H16O8	289.09182				7.52 * 10-6	5.92 * 10-6	4.89 * 10-6
**130**	Xanthoxic acid	C15H22O4	289.14103	1.81 * 10-4					
**132**		C12H26O5	289.14104	1.81 * 10-6	1.74 * 10-6	2.07 * 10-6			
**133**	Arginylasparagine	C10H20N6O4	289.16217	1.73 * 10-5	1.71 * 10-6				
**134**		C8H20NO8P	290.09991	3.16 * 10-6	3.53 * 10-6	3.08 * 10-6			
**135**		C9H23O8P	291.12030	3.73 *	3.68 * 10-	3.53 *			

				10-6	6	10-6			
**136**		C16H28O3	291.19308	1.98 * 10-6	2.65 * 10-6	2.72 * 10-6			
**137**	Oxo-octadecatetraenoic acid	C18H26O3	291.19547				1.86 * 10-2	1.71 * 10-2	1.54 * 10-2
**138**		C7H5N3O4S3	291.95124	4.04 * 10-7	2.81 * 10-7	2.35 * 10-7	3.18 * 10-7	2.51 * 10-7	2.17 * 10-7
**139**	N-Propionylprocainamide	C16H25N3O2	292.20130				1.61 * 10-5	1.70 * 10-5	
**140**		C8H21O9P	293.09957	2.39 * 10-6	2.40 * 10-6	2.05 * 10-6			
**141**	Histidylhistidine	C12H16N6O3	293.13597	6.20 * 10-6	6.56 * 10-5	7.22 * 10-4	3.29 * 10-3	2.57 * 10-6	2.86 * 10-6
**142**	N-Deoxy-1-fructosyl leucine	C12H23NO7	294.15475			1.74 * 10-5	2.57 * 10-6	2.59 * 10-6	2.75 * 10-6
**143**	Tridecanoylglycine	C15H29NO3	294.20396	5.96 * 10-4	4.50 * 10-1	5.29 * 10-2	1.94 * 10-2		
**144**		C7H4N4O2S3	294.93889	8.78 * 10-7	6.45 * 10-7	5.53 * 10-7			
**145**		C13H20O6	295.11522	8.60 * 10-6	8.43 * 10-6	9.54 * 10-6	5.06 * 10-6	4.18 * 10-6	4.30 * 10-6
**146**	Puromycin aminonucleoside	C12H18N6O3	295.15162	6.92 * 10-6	6.41 * 10-6	5.77 * 10-6	4.39 * 10-3	3.34 * 10-6	3.46 * 10-6
**147**	Hydroxylinolenic acid	C18H30O3	295.22677				3.93 * 10-2	4.10 * 10-1	3.51 * 10-2
**148**		C15H31NO3	296.21964	5.72 * 10-6	6.27 * 10-6	6.49 * 10-6	5.29 * 10-6	5.15 * 10-6	4.72 * 10-6

#	Putative Annotation[a]	Molecular Formula	Theo. m/z[b]	HA1[c]	HA2[c]	HA3[c]	Org1[c]	Org2[c]	Org3[c]
**149**		C17H29NO3	296.22207				1.93 * 10-6	1.80 * 10-6	1.95 * 10-6
**150**		C18H33NO2	296.25843				1.17 * 10-7	1.37 * 10-7	1.53 * 10-7
**151**		C11H20O5S2	297.08244				1.76 * 10-6	1.98 * 10-6	1.67 * 10-6
**152**	Deoxynivalenol	C15H20O6	297.13328				1.94 * 10-6	1.75 * 10-6	2.12 * 10-4
**153**	Dodecanoyl-sn-glycerol	C15H30O4	297.20363	1.12 * 10-5	1.50 * 10-1	1.60 * 10-1	1.75 * 10-2	1.58 * 10-2	1.87 * 10-2
**154**		C16H34O3	297.24004	7.94 * 10-6	8.15 * 10-6	8.38 * 10-6	2.09 * 10-6		1.67 * 10-6
**155**	Hydroxy-linoleic acid	C18H32O3	297.24242				5.61 * 10-2	5.97 * 10-2	5.94 * 10-2
**156**	Phytol	C20H40O	297.31519				1.71 * 10-2	1.82 * 10-2	
**157**	Palmitoleoylethanolamide	C18H35NO2	298.27408				4.23 * 10-6	5.60 * 10-7	6.46 * 10-7
**158**	Aminoguanosine	C10H14N6O5	299.11014	2.33 * 10-4	2.72 * 10-6	2.40 * 10-6			
**159**	Toxin T2 tetrol	C15H22O6	299.14894				2.02 * 10-6	0.00E+00	1.76 * 10-6
**160**		C12H27O6P	299.16179	1.30 * 10-7	1.24 * 10-7	1.26 * 10-7	5.72 * 10-6	5.72 * 10-6	5.28 * 10-6
**161**		C16H26O3S	299.16757				4.12 * 10-6	3.52 * 10-6	3.25 * 10-6
**162**	Methyl stearic acid	C19H38O2	299.29446				3.12 * 10-2	4.22 * 10-2	5.30 * 10-1
**163**		C12H29NO7	300.20169					2.00 * 10-6	1.90 * 10-6
**164**	Non-2-enoylcarnitine	C16H29NO4	300.21695	5.86 * 10-5	5.86 * 10-6	6.52 * 10-6	6.12 * 10-5	5.44 * 10-6	5.74 * 10-6
**165**	Sphingosine	C18H37NO2	300.28971	3.72 * 10-4	4.64 * 10-2	3.73 * 10-2	7.10 * 10-1	1.90 * 10-1	1.33 * 10-2
**166**		C8H14N4O7	301.07529	1.69 * 10-7	1.82 * 10-7	1.73 * 10-7	8.20 * 10-6	7.66 * 10-6	8.28 * 10-6
**167**	α-tocopheronolactone	C16H22O4	301.14103	4.57 * 10-3	4.21 * 10-2	4.70 * 10-1	1.99 * 10-2	1.87 * 10-2	2.00E+00
**168**	Panaxytriol	C17H26O3	301.17742	1.18 * 10-5					
**169**		C12H29O6P	301.17744	1.18 * 10-7	1.20 * 10-7	1.17 * 10-7	4.55 * 10-6	4.97 * 10-6	4.62 * 10-6
**170**		C15H28NO3P	302.18792	2.48 * 10-6	2.56 * 10-6	2.81 * 10-6	2.74 * 10-6	2.33 * 10-6	2.41 * 10-6
**171**		C10H27N3O7	302.19204	3.86 * 10-6	4.05 * 10-6	4.08 * 10-6	3.74 * 10-6	3.52 * 10-6	3.18 * 10-6
**172**	Hydroxy-cis-5-octenoylcarnitine	C15H27NO5	302.19622	1.17 *	1.62 * 10-	1.79 *	1.15 * 10-	1.64 *	1.38 *

				10-6	7	10-7	8	10-6	10-7
**173**	Phaseic acid	C15H20O5	303.12029	3.45 * 10-5	2.65 * 10-2	2.92 * 10-2			
**174**		C11H27O7P	303.15671	2.07 * 10-6	1.75 * 10-6				
**175**		C14H30N4OS	303.22113				6.00 * 10-6	7.49 * 10-6	7.90 * 10-6
**176**	MG(0:0/14:0/0:0)	C17H34O4	303.25299				3.44 * 10-2	3.17 * 10-2	3.47 * 10-2
**177**		C21H37N	304.29990	1.65 * 10-8	1.74 * 10-8	1.73 * 10-8	3.67 * 10-8	3.47 * 10-8	3.39 * 10-8
**178**	3-O-methyl-epicatechin	C16H16O6	305.10316	1.68 * 10-5		1.75 * 10-6	2.11 * 10-4		1.60 * 10-6
**179**	N4-Acetylsulfamonomethoxine	C13H14N4O3S	307.08532	5.29 * 10-6	4.87 * 10-5	4.83 * 10-5	3.91 * 10-6	3.94 * 10-5	3.62 * 10-6
**180**		C9H23O9P	307.11523	3.23 * 10-6	3.65 * 10-6	3.45 * 10-6			
**181**	β-D-Glucopyranosyloxy-3-octanone	C14H26O7	307.17395	2.89 * 10-5	2.82 * 10-6	2.49 * 10-5	3.89 * 10-6	2.89 * 10-6	2.95 * 10-5
**182**	N-Ethylcarboxamidoadenosine	C12H16N6O4	309.13088	8.43 * 10-6	7.66 * 10-6	7.76 * 10-6	3.98 * 10-5	3.85 * 10-4	3.90 * 10-5
**183**	Lauroyl diethanolamide	C16H33NO3	310.23528	1.53 * 10-7	1.47 * 10-7	1.42 * 10-7			
**184**	N-Hexadecanoylpyrrolidine	C20H39NO	310.31047				4.27 * 10-5	4.54 * 10-5	5.57 * 10-6
**185**	Karwinaphthol B	C17H20O4	311.12538	3.62 * 10-5					

#	Putative Annotation[a]	Molecular Formula	Theo. m/z[b]	HA1[c]	HA2[c]	HA3[c]	Org1[c]	Org2[c]	Org3[c]
**186**		C10H20N6O2S	311.12636	3.62 * 10-7	3.39 * 10-7	3.26 * 10-7			
**187**		C14H24O6	311.14653	1.02 * 10-7	1.10 * 10-7	1.09 * 10-7	4.67 * 10-6	4.02 * 10-6	4.10 * 10-6
**188**	Hydroxysterculic acid	C19H34O3	311.25810				2.33 * 10-6	2.52 * 10-6	2.59 * 10-6
**189**	Methionyl-Tyrosine	C14H20N2O4S	313.12215	1.82 * 10-6	1.92 * 10-5				
**190**	N-Undecylbenzenesulfonic acid	C17H28O3S	313.18320	1.75 * 10-5	2.60 * 10-6	3.89 * 10-4			
**191**	Oxo-nonadecanoic acid	C19H36O3	313.27372	3.81 * 10-5			4.22 * 10-7	4.04 * 10-7	3.98 * 10-7
**192**	Gingerdione	C17H24O4	315.15668	4.17 * 10-5	3.26 * 10-2	3.71 * 10-2			
**193**	Octanol glucoside	C14H28O6	315.17782	2.26 * 10-5	2.95 * 10-4	2.18 * 10-6			
**194**	Octadecanedioic acid	C18H34O4	315.25299				1.67 * 10-2	1.82 * 10-2	1.68 * 10-2
**195**	Hydroxypristanic acid	C19H38O3	315.28940				2.97 * 10-6	5.18 * 10-6	5.65 * 10-5
**196**	Oct-6-enedioylcarnitine	C15H25NO6	316.17550	3.36 * 10-5	3.56 * 10-6	3.36 * 10-6	4.76 * 10-6	4.71 * 10-5	4.49 * 10-6
**197**	Hydroxynon-2-enoylcarnitine	C16H29NO5	316.21188	3.19 * 10-6	3.30 * 10-5	3.26 * 10-7	2.97 * 10-7	2.84 * 10-7	2.77 * 10-7
**198**	Gibberellin A9	C19H24O4	317.17474	1.98 * 10-5	3.41 * 10-2	3.26 * 10-2			
**199**		C12H29N3S2	318.14365	2.93 * 10-6	2.64 * 10-6	3.35 * 10-6			
**200**		C12H21N7O2	318.16493				3.85 * 10-6	4.49 * 10-6	3.97 * 10-6
**201**	Suberoyl-L-carnitine	C15H27NO6	318.19113				3.21 * 10-5	3.44 * 10-6	3.85 * 10-5
**202**	N-methyl arachidonoyl amine	C21H35NO	318.27914	5.47 * 10-5			1.47 * 10-7	1.35 * 10-7	1.28 * 10-7
**203**	α-tocopheronic acid	C16H24O5	319.15159	2.20 * 10-3					
**204**		C11H27O8P	319.15163	2.00 * 10-6	2.37 * 10-6	2.08 * 10-6			
**205**		C13H28O7	319.17274	3.34 * 10-6	3.10 * 10-6	3.24 * 10-6			
**206**		C21H37NO	320.29482	5.96 * 10-6	5.90 * 10-6	7.01 * 10-6	1.45 * 10-7	1.42 * 10-7	1.39 * 10-7
**207**	Keto stearic acid	C18H34O3	321.24002	9.83 * 10-5	1.12 * 10-2	1.13 * 10-2	7.95 * 10-2	7.46 * 10-2	7.51 * 10-2
**208**		C8H10N4O6S2	323.01145	3.61 * 10-6	1.73 * 10-6				
**209**	Isococculidine	C18H23NO2	324.13604				1.85 * 10-	2.19 *	

							2	10-2	
**210**	Fructofuranose-dianhydride	C12H20O10	325.11292	4.12 * 10-5	2.50 * 10-1	2.84 * 10-2	2.37 * 10-2	1.78 * 10-2	1.47 * 10-2
**211**	Lactapiperanol D	C18H28O5	325.20097				1.95 * 10-6	1.80 * 10-5	1.62 * 10-6
**212**		C22H47N	326.37815	4.58 * 10-6	4.49 * 10-6	4.55 * 10-6	1.04 * 10-7	9.85 * 10-6	8.62 * 10-6
**213**		C14H24O7	327.14145	7.55 * 10-6	6.62 * 10-6	5.70 * 10-6	3.85 * 10-6	3.72 * 10-6	3.48 * 10-6
**214**	Alkaloid	C19H22N2O3	327.17140	4.75 * 10-6	4.09 * 10-6	5.10 * 10-6	6.21 * 10-6		
**215**	Calcium octanoate	C16H30CaO4	327.18430		1.98 * 10-6	2.31 * 10-6			
**216**	Dodecylbenzenesulfonic acid	C18H30O3S	327.19886		2.43 * 10-5	2.83 * 10-5	1.33 * 10-5	1.17 * 10-6	1.14 * 10-7
**217**	Acetoxy-2-hydroxy-16-heptadecen-4-one	C19H34O4	327.25302				1.83 * 10-5	2.26 * 10-6	1.85 * 10-5
**218**		C20H38O3	327.28942					1.85 * 10-6	1.72 * 10-6
**219**	Cymorcin monoglucoside	C16H24O7	329.15950				1.80 * 10-6		1.92 * 10-6
**220**	Methylgingerol	C18H28O4	331.18800	7.27 * 10-6	7.37 * 10-5	6.72 * 10-6	3.18 * 10-5	3.25 * 10-6	3.20 * 10-6
**221**	Trihydroxy-9-octadecenoic acid	C18H34O5	331.24790	1.78 * 10-5	1.97 * 10-2	2.36 * 10-2			
**222**		C25H17N	332.14336				2.71 * 10-6	2.65 * 10-6	3.19 * 10-6

#	Putative Annotation[a]	Molecular Formula	Theo. m/z[b]	HA1[c]	HA2[c]	HA3[c]	Org1[c]	Org2[c]	Org3[c]
**223**	Methyloctanedioylcarnitine	C16H29NO6	332.20679				2.20 * 10-5	1.73 * 10-6	2.27 * 10-4
**224**		C23H41N	332.33120	6.16 * 10-7	5.91 * 10-7	5.99 * 10-7	1.20 * 10-8	1.13 * 10-8	1.12 * 10-8
**225**		C9H4N2O8S2	332.94818	6.42 * 10-6	4.99 * 10-6	5.02 * 10-6			
**226**	Gingerol	C17H26O4	333.14627	3.92 * 10-3	4.40 * 10-1	4.17 * 10-2			
**227**		C17H26O5	333.16727	2.97 * 10-7	2.97 * 10-7	2.88 * 10-7	1.38 * 10-7	1.34 * 10-7	1.32 * 10-7
**228**	Gibberellin A20	C19H24O5	333.16965	3.27 * 10-5	2.25 * 10-2	2.99 * 10-2			
**229**		C25H19N	334.15899				5.19 * 10-6	6.38 * 10-6	6.30 * 10-6
**230**	Hydroxyoctanedioylcarnitine	C15H27NO7	334.18606				1.74 * 10-6	1.75 * 10-4	1.73 * 10-5
**231**		C16H31NO6	334.22244	1.10 * 10-7	1.07 * 10-7	1.07 * 10-7	2.64 * 10-6	2.45 * 10-6	2.86 * 10-6
**232**		C17H25N3O4	336.19182				2.31 * 10-6	2.13 * 10-6	2.16 * 10-6
**233**	Propanol, 1-methylethylamino-tripropylphenoxy	C21H37NO2	336.28974				1.86 * 10-6	1.68 * 10-6	1.58 * 10-6
**234**	Hydroxymugineic acid	C12H20N2O9	337.12579	3.47 * 10-5	3.31 * 10-6	3.45 * 10-6	1.72 * 10-6		1.88 * 10-6
**235**	Hydroxysphingosine	C18H37NO3	338.26656	6.63 * 10-5	6.52 * 10-2	6.31 * 10-2			
**236**	Docosenamide	C22H43NO	338.34176	2.59 * 10-5	3.43 * 10-5	5.27 * 10-5	3.96 * 10-7	7.35 * 10-7	1.11 * 10-8
**237**	Hydroxy-8-nor-2-fenchanone glucoside	C15H24O7	339.14144	7.25 * 10-6	6.83 * 10-5	6.95 * 10-6	4.47 * 10-5	3.28 * 10-5	3.25 * 10-5
**238**	Licochalcone A	C21H22O4	339.15766	6.98 * 10-5	1.90 * 10-6	1.59 * 10-6			
**239**		C21H38O3	339.28940				3.77 * 10-7	3.78 * 10-7	3.63 * 10-7
**240**		C23H49N	340.39379	2.52 * 10-6	2.42 * 10-6	2.53 * 10-6			
**241**	β-D-glucosaminyl-1-4-beta-D-glucosamine	C12H24N2O9	341.15710	5.61 * 10-6	5.73 * 10-5	5.94 * 10-6	3.97 * 10-6	3.72 * 10-5	3.27 * 10-6
**242**	Glycyl-l-histidyl-l-lysine	C14H24N6O4	341.19348	3.13 * 10-4	2.84 * 10-6	2.62 * 10-3			
**243**		C19H32O3S	341.21452				7.64 * 10-6	6.37 * 10-6	6.31 * 10-6
**244**		C18H38O4	341.26626	1.20 * 10-7	1.10 * 10-7	1.22 * 10-7	2.15 * 10-6	2.17 * 10-6	
**245**	Oxo-heneicosanoic acid	C21H40O3	341.30502			2.29 * 10-2	1.90 * 10-1	1.25 * 10-2	1.13 * 10-2
**246**	Pentyl-3-1-naphthoyl indole	C24H23NO	342.18521				2.20 * 10-	2.17 *	2.62 *

							6	10-6	10-5
**247**	Sodium decylbenzenesulfonate	C16H25NaO3S	343.13144	6.50 * 10-5	6.94 * 10-5	6.29 * 10-5			
**248**	N-Amino-oxadiazol-yl-ethylimidazo-d-pyridinyl methylpiperidin-amine	C16H22N8O	343.19895	3.43 * 10-6	2.61 * 10-6	2.74 * 10-6			
**249**		C19H38N2O3	343.29554	2.27 * 10-7	2.56 * 10-7	2.56 * 10-7	1.37 * 10-7	1.26 * 10-7	1.19 * 10-7
**250**	Decenedioylcarnitine	C17H29NO6	344.20678	2.22 * 10-5	2.16 * 10-5	2.45 * 10-6			
**251**		C10H30N7O4P	344.21695	2.33 * 10-6	2.85 * 10-6	2.37 * 10-6			
**252**	N-propyl arachidonoyl amine	C23H39NO	346.31044	1.91 * 10-5	1.75 * 10-2	2.40 * 10-1	5.98 * 10-2	5.67 * 10-2	5.24 * 10-2
**253**	Mytilin B	C14H22N2O8	347.14651	2.35 * 10-6	2.19 * 10-6	2.84 * 10-6			
**254**	Arginyl-glycyl-aspartic acid	C12H22N6O6	347.16764	3.39 * 10-6	3.27 * 10-6	3.36 * 10-5			
**255**		C19H41NO4	348.31087				3.18 * 10-6	3.33 * 10-6	3.24 * 10-6
**256**		C23H41NO	348.32610	2.00 * 10-6	2.27 * 10-6	2.12 * 10-6	5.12 * 10-6	4.78 * 10-6	4.88 * 10-6
**257**	N-Carboxymethyl-hydroxysuccinoyl arginine	C12H20N4O8	349.13563	1.94 * 10-6	2.43 * 10-5	1.73 * 10-6			
**258**	Magnesium octanoate	C16H30MgO4	349.16217	3.89 * 10-6	3.29 * 10-6	3.49 * 10-6			
**259**	Hydroperoxy-octadecatrienoic acid	C18H30O5	349.19854	2.87 * 10-5	2.82 * 10-2	3.20 * 10-1			

#	Putative Annotation[a]	Molecular Formula	Theo. m/z[b]	HA1[c]	HA2[c]	HA3[c]	Org1[c]	Org2[c]	Org3[c]
**260**		C17H32O7	349.22240				6.43 * 10-6	9.18 * 10-6	9.45 * 10-6
**261**	Coutaric acid	C18H27N3O4	350.20679	3.84 * 10-5	3.59 * 10-6	3.59 * 10-6	4.32 * 10-4	4.12 * 10-5	3.88 * 10-5
**262**	Coumarin 6	C20H18N2O2S	351.11490	2.46 * 10-5	2.74 * 10-6	3.23 * 10-6		1.70 * 10-6	
**263**	trans-p-Menthane-1,7,8-triol 8-glucoside	C16H30O8	351.20016	3.65 * 10-6	3.77 * 10-6	3.44 * 10-6	4.14 * 10-6	3.51 * 10-6	3.36 * 10-6
**264**	Adamantylamide-alanyl-isoglutamine	C18H30N4O3	351.23807					2.23 * 10-6	2.73 * 10-4
**265**	MG(0:0/16:1(9Z)/0:0)	C19H36O4	351.25058	9.56 * 10-4	1.23 * 10-2	1.50 * 10-1	8.46 * 10-2	7.97 * 10-2	7.80 * 10-1
**266**		C22H41NO2	352.32103				6.87 * 10-6	7.24 * 10-6	6.80 * 10-6
**267**		C9H4O9S3	352.90907	3.01 * 10-6	2.03 * 10-6				
**268**		C19H38O4	353.26625	3.47 * 10-7	4.01 * 10-7	3.50 * 10-7	4.48 * 10-7	4.26 * 10-7	4.23 * 10-7
**269**	Ceriporic acid C	C21H36O4	353.26864				1.76 * 10-2	1.65 * 10-2	1.64 * 10-2
**270**		C22H43NO2	354.33668			1.71 * 10-6	5.56 * 10-6	5.59 * 10-6	6.26 * 10-6
**271**	Neryl glucoside	C16H28O6	355.15257	2.26 * 10-7	1.86 * 10-5	1.78 * 10-6			
**272**	Hydroxytridecane-1,2,3-tricarboxylate	C16H28O7	355.17272	3.25 * 10-5	2.92 * 10-2	2.69 * 10-2			
**273**	Ceriporic acid B	C21H38O4	355.28429				7.72 * 10-2	7.39 * 10-2	7.11 * 10-2
**274**	Tridecenoylcarnitine	C20H37NO4	356.27957				2.20 * 10-6	2.74 * 10-5	2.82 * 10-5
**275**	Amino-phenoxyphenylethylamino quinazoline	C22H20N4O	357.17189					1.57 * 10-6	1.55 * 10-6
**276**		C18H38O5	357.26117	7.22 * 10-6	8.40 * 10-6	7.78 * 10-6	4.90 * 10-6	4.17 * 10-6	4.02 * 10-6
**277**		C25H45N	360.36251	2.01 * 10-6		1.69 * 10-6	3.66 * 10-6	4.21 * 10-6	3.84 * 10-6
**278**		C26H21NO	364.16955				2.72 * 10-6	2.73 * 10-6	2.97 * 10-6
**279**	Hexose	C12H22O11	365.10543	2.15 * 10-5	8.77 * 10-2	6.79 * 10-2	1.30 * 10-1	7.45 * 10-2	6.31 * 10-2
**280**	Cyclopentylamino-8-methylaminopurin-9-yl-5-hydroxymethyloxolane-3,4-diol	C16H24N6O4	365.19348	3.68 * 10-5	3.87 * 10-6	3.73 * 10-5			
**281**	Eicosanedioic acid	C20H38O4	365.26623	1.79 * 10-5	2.70 * 10-1	3.29 * 10-2	1.89 * 10-2	2.10 * 10-1	
**282**		C26H23NO	366.18521				1.77 * 10-7	2.01 * 10-7	2.13 * 10-7
**283**	N-Acetylsphinganine	C20H41NO3	366.29789	2.45 *	2.18 * 10-	2.35 *			

				10-5	6	10-5			
**284**	Daphnoretin methyl ether	C20H14O7	367.08157	3.18 * 10-6	2.64 * 10-5	2.39 * 10-5	5.72 * 10-5	6.56 * 10-6	5.43 * 10-6
**285**		C18H32O6	367.20914	1.28 * 10-7	1.64 * 10-7	1.61 * 10-7	1.93 * 10-6	2.29 * 10-6	2.28 * 10-6
**286**	PGE2alpha dimethyl amine	C22H41NO3	368.31592				2.59 * 10-2	2.10 * 10-1	2.84 * 10-2
**287**		C25H53N	368.42510	6.17 * 10-6	5.34 * 10-6	5.57 * 10-6	2.73 * 10-6	2.56 * 10-6	2.44 * 10-6
**288**		C15H32N2S4	369.15205	2.10 * 10-6	2.00 * 10-6	2.54 * 10-6			
**289**	Mitraphylline	C21H24N2O4	369.18088	2.11 * 10-3	1.79 * 10-2	1.81 * 10-2		1.93 * 10-2	
**290**	Arginyl-prolyl-proline	C16H28N6O4	369.22478		2.67 * 10-6	2.43 * 10-6			
**291**		C27H44	369.35162				4.18 * 10-6	7.75 * 10-6	1.05 * 10-7
**292**		C23H48N2O	369.38397	9.63 * 10-6	8.55 * 10-6	8.56 * 10-6	3.70 * 10-6	3.84 * 10-6	3.63 * 10-6
**293**	cis-5-Tetradecenoylcarnitine	C21H39NO4	370.29519				2.15 * 10-2	2.20 * 10-1	1.96 * 10-2
**294**	Hydroxyyohimbine	C21H26N2O4	371.19763	7.57 * 10-6				6.72 * 10-6	6.34 * 10-6
**295**	Sativic acid	C18H36O6	371.24041	1.46 * 10-5	1.55 * 10-2	1.39 * 10-2			
**296**		C21H41NO4	372.31088				9.83 * 10-6	1.10 * 10-7	1.12 * 10-7

#	Putative Annotation[a]	Molecular Formula	Theo. m/z[b]	HA1[c]	HA2[c]	HA3[c]	Org1[c]	Org2[c]	Org3[c]
**297**		C22H45NO3	372.34725		1.76 * 10-6		2.82 * 10-6	2.41 * 10-6	2.74 * 10-6
**298**	Tetrahydrodeoxycorticosterone	C21H34O3	373.21327	2.18 * 10-6	2.67 * 10-6	2.76 * 10-6	3.01 * 10-6	2.70 * 10-6	2.38 * 10-6
**299**	Deoxyoleandolide	C20H36O6	373.25847	1.68 * 10-5					
**300**		C25H27NO2	374.21143				7.02 * 10-6	7.49 * 10-6	8.15 * 10-6
**301**		C21H43NO4	374.32651				4.57 * 10-6	5.89 * 10-6	5.44 * 10-6
**302**	Picrasin B	C21H28O6	377.19587	5.91 * 10-5	5.61 * 10-2	4.47 * 10-2			
**303**		C20H38N2OS	377.25976				3.21 * 10-6	3.83 * 10-6	3.24 * 10-6
**304**	Pentosidine	C17H26N6O4	379.20883	1.93 * 10-4	1.90 * 10-1	2.16 * 10-2			
**305**		C20H40N2OS	379.27534				1.65 * 10-6	1.82 * 10-6	1.80 * 10-6
**306**	MG(0:0/18:1(11Z)/0:0)	C21H40O4	379.28188	9.62 * 10-5	1.40 * 10-1	1.40 * 10-1	4.98 * 10-2	4.94 * 10-2	4.78 * 10-2
**307**		C20H42O6	379.30544	3.45 * 10-6	3.00 * 10-6	3.55 * 10-6	6.96 * 10-6	6.16 * 10-6	6.67 * 10-6
**308**	Arabinosylamino-glucosylamino-propanenitrile	C14H25N3O9	380.16448				1.78 * 10-5	2.72 * 10-6	2.66 * 10-6
**309**	N-palmitoyl threonine	C20H39NO4	380.27713		1.77 * 10-2	2.59 * 10-2	2.46 * 10-2	2.18 * 10-2	2.80 * 10-1
**310**	Prenylcatechin	C20H22O6	381.13086	1.99 * 10-4					
**311**		C15H25O9P	381.13091	1.99 * 10-6	1.81 * 10-6	1.78 * 10-6			
**312**	Egtazic acid	C14H24N2O10	381.15201	2.85 * 10-5	1.75 * 10-5	1.76 * 10-6			
**313**	Azido-methyl-dioxopyrimidin-yl oxolanyl methyl-amino-methylpentanoate	C16H24N6O5	381.18838	4.89 * 10-6	4.68 * 10-6	4.61 * 10-6	2.75 * 10-6	2.25 * 10-6	2.55 * 10-6
**314**	MG(0:0/18:0/0:0)	C21H42O4	381.29753	7.98 * 10-4			9.83 * 10-6	1.07 * 10-7	1.09 * 10-7
**315**		C26H23NO2	382.18012					2.00 * 10-6	1.75 * 10-6
**316**		C16H27N7O4	382.21973	5.44 * 10-6	6.02 * 10-6				
**317**	Enterodiol sulfate	C18H22O7S	383.11604				6.47 * 10-6	3.27 * 10-4	2.36 * 10-5
**318**		C24H27NO2	384.19342	2.08 * 10-7	3.09 * 10-7	3.45 * 10-7	2.96 * 10-6	2.56 * 10-6	2.71 * 10-6
**319**		C23H45NO3	384.34724		1.66 * 10-6		5.96 * 10-6	6.55 * 10-6	7.59 * 10-6
**320**		C18H36N6O3	385.29246	1.71 * 10-7	1.77 * 10-7	1.71 * 10-7	3.49 * 10-6	2.74 * 10-6	2.82 * 10-6

**321**	Vitamin D3	C27H44O	385.34649		2.71 * 10-2	3.14 * 10-2	2.52 * 10-2	2.57 * 10-2	2.30 * 10-1
**322**		C23H48N2O2	385.37888	4.84 * 10-6	4.33 * 10-6	4.18 * 10-6	1.74 * 10-6	2.63 * 10-6	2.31 * 10-6
**323**	Glycerol trihexanoate	C21H38O6	387.27412		2.42 * 10-6	2.56 * 10-6			
**324**		C25H25NO3	388.19069				4.70 * 10-6	4.96 * 10-6	5.28 * 10-6
**325**		C12H34N7O5P	388.24316	4.56 * 10-6	3.67 * 10-6	4.36 * 10-6			
**326**		C22H45NO4	388.34215	2.35 * 10-6	2.15 * 10-6	2.22 * 10-6			
**327**	L-Pyroglutamyl-L-histidyl-3,3-dimethylprolinamide	C18H26N6O4	391.20912	2.88 * 10-6	2.49 * 10-5	3.37 * 10-6			
**328**	N-palmitoyl isoleucine	C22H43NO3	392.31351	7.15 * 10-3	5.86 * 10-2	5.58 * 10-2	3.24 * 10-2	3.30 * 10-1	3.62 * 10-2
**329**	Glycylphenylalanylleucylglycine	C19H28N4O5	393.21326		2.14 * 10-5	2.35 * 10-5	2.42 * 10-4	2.69 * 10-5	2.68 * 10-6
**330**	Docosanedioic acid	C22H42O4	393.29753	2.74 * 10-5	5.30 * 10-1		3.34 * 10-2	3.41 * 10-2	3.90 * 10-1
**331**		C21H42N2O3	393.30878	7.92 * 10-6	8.46 * 10-6	7.51 * 10-6			
**332**	Apiosyllotaustralin	C16H27NO10	394.16921	2.28 * 10-5	1.80 * 10-6				
**333**	Tylophorine	C24H27NO4	394.20296		1.69 * 10-6	1.93 * 10-5			

#	Putative Annotation[a]	Molecular Formula	Theo. m/z[b]	HA1[c]	HA2[c]	HA3[c]	Org1[c]	Org2[c]	Org3[c]
**334**		C24H46N2O2	395.36323	2.16 * 10-6	2.60 * 10-6	2.54 * 10-6	4.97 * 10-6	4.52 * 10-6	4.54 * 10-6
**335**	Etarotene	C25H32O2S	397.21972	1.98 * 10-6	2.80 * 10-6	2.29 * 10-6			
**336**	Benzoyl arginine nitroanilide	C19H22N6O4	399.17878	2.48 * 10-5	1.94 * 10-6	1.79 * 10-6			
**337**	Hexanoyl-alpha-glucopyranose	C18H32O8	399.19894	3.43 * 10-4					
**338**		C23H46N2O3	399.35813	5.66 * 10-6	5.85 * 10-6	5.84 * 10-6	2.42 * 10-6	1.96 * 10-6	2.55 * 10-6
**339**		C21H37NO4S	400.25164				2.55 * 10-6	2.53 * 10-6	1.60 * 10-6
**340**	Palmitoylcarnitine	C23H45NO4	400.34214	4.57 * 10-4	3.86 * 10-2	3.48 * 10-2		1.55 * 10-2	1.56 * 10-2
**341**		C19H31NO8	402.21227				1.06 * 10-7	9.40 * 10-6	9.19 * 10-6
**342**		C23H47NO4	402.35781	6.56 * 10-7	6.22 * 10-7	6.18 * 10-7	1.46 * 10-7	1.42 * 10-7	1.42 * 10-7
**343**		C11H32N7O7P	406.21738				2.06 * 10-6	2.80 * 10-6	1.77 * 10-6
**344**	Adenosine, butylamino-N-cyclopentyl	C19H30N6O4	407.24042	2.24 * 10-6	2.39 * 10-6	1.95 * 10-6			
**345**		C22H46O6	407.33675	3.34 * 10-6	3.74 * 10-6	4.14 * 10-6	3.13 * 10-6	2.74 * 10-6	2.27 * 10-6
**346**		C28H25NO2	408.19577				6.02 * 10-6	4.66 * 10-6	4.66 * 10-6
**347**	Pentadecanoylcarnitine	C22H43NO4	408.30845	8.16 * 10-6	2.36 * 10-5	3.38 * 10-7	3.92 * 10-7	5.55 * 10-5	6.36 * 10-7
**348**	Met-leu-phe	C20H31N3O4S	410.21138				2.42 * 10-5	1.69 * 10-5	
**349**	Dimethoxypyridin-yl-ethoxy-methoxy-hexahydrophenanthridinol	C23H28N2O5	413.20715		5.29 * 10-6	4.26 * 10-6	2.55 * 10-6	3.44 * 10-6	1.91 * 10-6
**350**		C24H38O4	413.26625	1.22 * 10-7	1.94 * 10-7	2.36 * 10-7	4.42 * 10-6	4.10 * 10-6	4.26 * 10-6
**351**	MG(0:0/20:3(8Z,11Z,14Z/0:0)_	C23H40O6	413.28977			1.99 * 10-5	1.63 * 10-5		
**352**	MG(0:0/20:5(5Z,8Z,11Z,14Z,17Z)/0:0)	C23H36O4	415.22452			5.54 * 10-2			4.88 * 10-2
**353**	Amino-imidazolyl-propanoyl-benzamidoethylamino-methylpentanoic acid	C21H29N5O4	416.22791	2.13 * 10-7	2.27 * 10-7	2.24 * 10-7	2.46 * 10-7	2.41 * 10-7	2.27 * 10-7
**354**		C24H49NO4	416.37348	4.33 * 10-6		3.89 * 10-6	1.64 * 10-6		
**355**	CPA_18:2_9Z,12Z_0:0_	C21H37O6P	417.23945	1.67 * 10-6				1.58 * 10-6	1.59 * 10-6
**356**	Arachidonoylglycerol	C23H38O4	417.24017	1.67 * 10-4				1.58 * 10-2	1.59 * 10-2
**357**	Phenylalanyl-prolyl-arginine	C20H30N6O4	419.24038	2.47 *	1.95 * 10-				

				10-5	5				
**358**		C22H40O6	423.27173	8.33 * 10-6	1.66 * 10-7	1.90 * 10-7	1.76 * 10-6		1.60 * 10-6
**359**	Calcidiol	C27H44O2	423.32335		3.10 * 10-1	3.59 * 10-2			
**360**	Hydroxymelatonin glucuronide	C19H24N2O9	425.15708	1.87 * 10-5	2.57 * 10-6	2.26 * 10-6			
**361**	Taraxinic acid glucosyl ester	C21H28O9	425.18196	1.86 * 10-6	1.79 * 10-5		4.52 * 10-6	3.57 * 10-5	4.25 * 10-5
**362**		C30H32O2	425.24785				3.23 * 10-6	5.02 * 10-6	6.89 * 10-6
**363**		C22H49O5P	425.33899	1.94 * 10-6	3.70 * 10-6	3.85 * 10-6			
**364**	δ-Tocopherol	C27H46O2	425.33900	1.94 * 10-5					
**365**		C27H56N2O	425.44655	7.16 * 10-6	6.56 * 10-6	5.92 * 10-6			1.60 * 10-6
**366**	Mespirenone	C25H30O4S	427.19388	2.46 * 10-5	2.81 * 10-5	2.76 * 10-6			
**367**	Caesalpinin N	C23H32O6	427.20911	3.31 * 10-5	5.70 * 10-1	4.76 * 10-2			
**368**	Dihydroxyprostaglandin F1a	C20H36O7	427.21009	3.31 * 10-6	5.78 * 10-4	4.76 * 10-6			
**369**	Leupeptin	C20H38N6O4	427.30273	2.22 * 10-3	2.73 * 10-2	3.18 * 10-2			
**370**		C24H42O6	427.30541			2.41 * 10-6	1.84 * 10-6	1.75 * 10-6	

#	Putative Annotation[a]	Molecular Formula	Theo. m/z[b]	HA1[c]	HA2[c]	HA3[c]	Org1[c]	Org2[c]	Org3[c]
**371**		C25H50N2O3	427.38942	2.68 * 10-6	2.53 * 10-6	1.74 * 10-6			
**372**	Valyl-prolyl-glycyl-valyl-glycine	C19H33N5O6	428.24904				1.84 * 10-6	1.79 * 10-5	1.68 * 10-5
**373**	Stearoylcarnitine	C25H49NO4	428.37344	1.10 * 10-1			2.31 * 10-6	1.90 * 10-6	1.90 * 10-6
**374**		C20H40N6O4	429.31867	2.22 * 10-7	2.21 * 10-7	2.25 * 10-7	5.52 * 10-6	4.21 * 10-6	4.73 * 10-6
**375**	Hexadecanedioic acid mono-carnitine ester	C23H43NO6	430.31634				1.62 * 10-6	1.88 * 10-5	1.92 * 10-6
**376**		C25H51NO4	430.38910	4.35 * 10-7	4.02 * 10-7	3.78 * 10-7	8.66 * 10-6	8.92 * 10-6	8.14 * 10-6
**377**		C18H41NO10	432.28034				7.33 * 10-6	7.11 * 10-6	7.18 * 10-6
**378**	Dimethoxyphenyl-2-hydroxyethylamino-7-2-methoxyethyl-1,3-dimethylpurine-2,6-dione	C20H27N5O6	434.20176				2.40 * 10-5	2.14 * 10-6	2.53 * 10-6
**379**	Tyrosyl-prolyl-arginine	C20H30N6O5	435.23533	2.32 * 10-6	2.09 * 10-6	2.89 * 10-6			
**380**		C13H39N2O6PS2	437.18801	2.20 * 10-6		1.70 * 10-6			
**381**		C24H30O6	437.19348	1.63 * 10-7	1.64 * 10-7	1.56 * 10-7	8.35 * 10-6	7.82 * 10-6	8.11 * 10-6
**382**	Val-Arg-Tyr	C20H32N6O5	437.25099	2.29 * 10-5	2.13 * 10-5	2.73 * 10-5			
**383**	Pangamic acid	C20H40N2O8	437.28737		3.45 * 10-5	3.68 * 10-6			
**384**	Calcitriol	C27H44O3	439.31827	2.50 * 10-4	2.68 * 10-2	3.29 * 10-2			
**385**	Glyceryl diacetate 1-oleate	C25H44O6	441.32110				2.64 * 10-5	2.14 * 10-4	1.74 * 10-6
**386**		C24H44N2O5	441.33231	3.14 * 10-6	3.24 * 10-6	3.82 * 10-6	1.76 * 10-6	2.16 * 10-6	1.81 * 10-6
**387**	Tyrosyl-glycyl-glycyl-phenylalanyl	C22H26N4O6	443.19253	2.39 * 10-5	4.23 * 10-5	3.56 * 10-6	4.37 * 10-5	4.45 * 10-6	4.28 * 10-6
**388**	PG(6:0/6:0)	C18H35O10P	443.20406	3.33 * 10-3	2.84 * 10-2	2.50 * 10-1			
**389**	Carboxyethylthio-phenyloctyl phenyl Propanoic acid	C26H34O4S	443.22519	2.34 * 10-4			1.70 * 10-5		
**390**		C22H38N2O7	443.27516	1.83 * 10-6	1.85 * 10-6		1.71 * 10-6		
**391**	Ascorbyl stearate	C24H42O7	443.30034				3.23 * 10-5	2.69 * 10-6	2.97 * 10-5
**392**		C24H28O8	445.18572				2.34 * 10-6	2.17 * 10-6	2.03 * 10-6
**393**		C22H46O7	445.31359	1.87 * 10-7	1.86 * 10-7	1.91 * 10-7	1.31 * 10-7	1.25 * 10-7	1.19 * 10-7
**394**		C23H48N4O4	445.37485			2.53 *	3.38 * 10-	4.50 *	5.72 *

						10-6	6	10-6	10-6
**395**	Azetidinecarboxylic acid	C18H31N7O5	448.22792	1.39 * 10-7	1.37 * 10-7	1.43 * 10-7		5.23 * 10-6	3.71 * 10-6
**396**		C18H33N7O5	450.24354	2.51 * 10-6	3.37 * 10-6	3.29 * 10-6			
**397**		C16H43N5O3S3	450.26011				1.83 * 10-6	1.58 * 10-6	
**398**		C12H31N14O3P	451.25136	2.10 * 10-6	2.18 * 10-6				
**399**	Chaksine	C22H38N6O4	451.30273		2.38 * 10-2	4.74 * 10-2			
**400**	Sorbitan stearate	C24H46O6	453.31868		2.00 * 10-4	2.66 * 10-5			
**401**		C26H48N2O4	453.36870	1.64 * 10-6	1.97 * 10-6	1.75 * 10-6	4.13 * 10-6	2.99 * 10-6	3.62 * 10-6
**402**	Hydroxyhexadecanoylcarnitine	C23H45NO5	454.29293				1.10 * 10-1	9.30 * 10-1	9.58 * 10-2
**403**	Oleoylglycerone phosphate	C21H39O7P	457.23256	1.32 * 10-5		1.35 * 10-2	4.20 * 10-1	4.18 * 10-2	4.73 * 10-2
**404**	Lyso-arachidonoyl-phosphatidate	C23H39O7P	459.25002	2.39 * 10-6	1.91 * 10-5	2.43 * 10-6	1.90 * 10-5	2.21 * 10-4	2.62 * 10-5
**405**	Gingerdiol-O-beta-glucopyranoside	C23H38O9	459.26003	5.12 * 10-6	5.89 * 10-4	5.28 * 10-6	3.97 * 10-5	4.13 * 10-6	3.66 * 10-6
**406**		C21H50N2S4	459.29285	1.93 * 10-6	2.33 * 10-6		2.18 * 10-6	2.06 * 10-6	1.74 * 10-6
**407**	Methylcysteine sulfoxide	C22H37NO5S2	460.21775	1.93 * 10-4	2.57 * 10-6	1.84 * 10-6	3.15 * 10-6	2.66 * 10-6	2.67 * 10-6

#	Putative Annotation[a]	Molecular Formula	Theo. m/z[b]	HA1[c]	HA2[c]	HA3[c]	Org1[c]	Org2[c]	Org3[c]
**408**		C13H33N14O3P	465.26703	1.79 * 10-6	1.71 * 10-6				
**409**	Diphenylmethyl-piperazinyl-hydroxypropoxy-1H-indole-2-carbonitrile	C29H30N4O2	467.24631	1.85 * 10-6	2.55 * 10-6	2.17 * 10-5			
**410**	Dukunolide D	C26H28O8	469.18337		1.92 * 10-6	1.75 * 10-6			1.52 * 10-6
**411**	Lucidenic acid	C27H34O7	471.23631	4.47 * 10-6	7.59 * 10-6	7.26 * 10-4			
**412**		C24H42N2O7	471.30649		1.93 * 10-6		1.76 * 10-6		1.88 * 10-6
**413**		C18H29N15O	472.27522				2.84 * 10-6	2.52 * 10-6	3.76 * 10-6
**414**	LysoPE(0:0/18:3(6Z,9Z,12Z))	C23H42NO7P	476.27717				4.60 * 10-1	4.79 * 10-2	4.26 * 10-2
**415**	Methyl cellulose	C20H38O11	477.23063	2.25 * 10-7	2.21 * 10-7	2.29 * 10-6	1.66 * 10-6	1.63 * 10-6	1.42 * 10-7
**416**	LysoPE(0:0/18:2(9Z,12Z))	C23H44NO7P	478.29138				3.75 * 10-6	1.63 * 10-6	3.46 * 10-6
**417**	2-Methoxy-estradiol-17b 3-glucuronide	C25H34O9	479.22521		1.97 * 10-6	1.71 * 10-6	2.20 * 10-6		1.90 * 10-6
**418**		C27H70N6	479.57383		1.87 * 10-6				
**419**	Hydroxy-octadecenoylcarnitine	C25H47NO5	480.30858				1.42 * 10-2	1.32 * 10-2	1.25 * 10-2
**420**	Oxo-androsterone glucuronide	C25H36O9	481.24212	1.83 * 10-6		1.93 * 10-5			
**421**		C20H42O11	481.26192	3.43 * 10-7	3.70 * 10-7	3.64 * 10-7	2.19 * 10-7	2.00 * 10-7	1.95 * 10-7
**422**	Neoandrographolide	C26H40O8	481.27716	1.68 * 10-6		1.91 * 10-6			
**423**	Crustecdysone	C27H44O7	481.31354	2.20 * 10-5	4.11 * 10-6	5.57 * 10-6			
**424**	Hydroxy-octadecanoylcarnitine	C25H49NO5	482.32423				3.18 * 10-2	3.26 * 10-2	3.20 * 10-1
**425**	β-Hydroxyandrosterone-3-glucuronide	C25H38O9	483.25646	2.05 * 10-6	2.45 * 10-6	2.38 * 10-6			
**426**	N-Docosahexaenoyl Arginine	C28H44N4O3	485.34782	2.13 * 10-5		2.11 * 10-5			
**427**		C10H11N6O7PS4	486.93823				4.94 * 10-6	4.87 * 10-6	4.65 * 10-6
**428**	Austalide I	C27H34O8	487.23320	1.89 * 10-6	2.27 * 10-6	2.13 * 10-6	4.84 * 10-6	3.54 * 10-5	2.38 * 10-5
**429**		C24H50O8	489.33980	1.95 * 10-7	1.85 * 10-7	1.91 * 10-7	1.22 * 10-7	1.21 * 10-7	1.20 * 10-7
**430**	Austalide F	C26H34O9	491.22680		1.72 * 10-3	2.93 * 10-5			

**431**	Epoxy-retinoyl-beta-D-glucuronide	C26H36O9	493.24088	1.73 * 10-6		1.92 * 10-5			
**432**		C32H48N2S	493.36111	1.86 * 10-6		1.81 * 10-6			1.92 * 10-6
**433**		C18H47N5O4S3	494.28644				2.28 * 10-6	1.85 * 10-6	2.26 * 10-6
**434**	Chenodeoxycholylthreonine	C28H47NO6	494.34527				1.79 * 10-6	2.81 * 10-5	2.22 * 10-6
**435**		C13H31N14O5P	495.24120	3.33 * 10-6	3.72 * 10-6	4.43 * 10-6			
**436**	Ethoxy-epoxy-hydroxy-cyathen-al -xyloside	C27H42O8	495.29285		1.68 * 10-6	2.29 * 10-5			
**437**		C26H9NO2S4	495.95891	5.93 * 10-6	6.82 * 10-6	6.89 * 10-6			
**438**	Dihydroxy-2-hydroxyocta-dien-1-yl cyclopentyl hept-5-enoylcarnitine	C27H45NO7	496.32857				4.47 * 10-5	3.48 * 10-5	3.87 * 10-6
**439**	Docosa-pentaenoyl carnitine	C29H47NO4	496.33973	2.53 * 10-4	3.10 * 10-1	3.29 * 10-2	8.32 * 10-2	8.40 * 10-1	7.79 * 10-2
**440**		C22H44O12	501.29054	1.28 * 10-7	1.27 * 10-7				
**441**		C19H54N2O2S5	503.28621	4.20 * 10-6	3.46 * 10-6	2.43 * 10-6	2.23 * 10-6	2.07 * 10-6	2.08 * 10-6
**442**	Germine	C27H43NO8	510.30614				1.97 * 10-2		1.71 * 10-2
**443**	LysoPE(0:0/20:0)	C25H52NO7P	510.35542				2.14 * 10-2	1.77 * 10-2	1.93 * 10-2
**444**	sialosyl-Tn saccharide	C19H32N2O14	513.19418		1.90 * 10-6	2.82 * 10-4			

#	Putative Annotation[a]	Molecular Formula	Theo. m/z[b]	HA1[c]	HA2[c]	HA3[c]	Org1[c]	Org2[c]	Org3[c]
**445**		C32H64O4	513.48766				1.78 * 10-6	1.92 * 10-6	2.03 * 10-6
**446**	Taurocholic acid	C26H45NO7S	516.30144				4.81 * 10-6	5.89 * 10-4	5.12 * 10-6
**447**	LysoPC(18:3(6Z,9Z,12Z))	C26H48NO7P	518.32412				5.88 * 10-2	4.67 * 10-2	6.45 * 10-2
**448**	LysoPC(0:0/18:2(9Z,12Z))	C26H50NO7P	520.33754					1.81 * 10-6	1.73 * 10-6
**449**	Chenodeoxycholylglutamic acid	C29H47NO7	522.34410				2.99 * 10-6	3.01 * 10-6	1.96 * 10-6
**450**	LysoPC(18:1(11Z))	C26H52NO7P	522.35542		1.79 * 10-2		3.89 * 10-2	3.63 * 10-2	3.46 * 10-2
**451**	Physangulide	C28H42O9	523.29125					2.13 * 10-6	1.94 * 10-5
**452**	Deoxocucurbitacin I	C30H44O6	523.30301	2.97 * 10-3					
**453**	LysoPC(18:0/0:0)	C26H54NO7P	524.37107				4.57 * 10-2	4.21 * 10-2	4.70 * 10-1
**454**		C22H46O12	525.28812	3.31 * 10-7	3.34 * 10-7	3.63 * 10-7	2.33 * 10-7	2.08 * 10-7	2.10 * 10-7
**455**	Phaseolus epsilon	C25H34O12	527.20988		2.94 * 10-6	1.83 * 10-6			
**456**	Territrem B	C29H34O9	527.22669	2.87 * 10-6	2.77 * 10-6				
**457**	Uscharidin	C29H38O9	531.25886		4.32 * 10-6		6.20 * 10-6		6.70 * 10-6
**458**		C25H58N2OS4	531.35048		1.94 * 10-6	2.40 * 10-6			
**459**		C26H54O9	533.36599	1.49 * 10-7	1.39 * 10-7	1.48 * 10-7	1.03 * 10-7	9.77 * 10-6	1.02 * 10-7
**460**	Octadecanoyloxy hexadecenoic acid	C34H64O4	537.48773				2.58 * 10-6	4.75 * 10-6	5.06 * 10-6
**461**	Octadecanoyloxy-hexadecanoic acid	C34H66O4	539.50350				2.18 * 10-6	3.52 * 10-6	4.25 * 10-6
**462**		C32H49NO4S	544.34543				2.47 * 10-6		2.43 * 10-6
**463**	PI(14:0/0:0)	C23H45O12P	545.27214	3.44 * 10-6	2.97 * 10-6	2.24 * 10-6			
**464**		C15H34N18O2S	553.27256	4.62 * 10-6	4.19 * 10-6	5.43 * 10-6	8.32 * 10-6		7.55 * 10-6
**465**		C23H47N3O6P2S	556.27323				4.34 * 10-6	3.64 * 10-6	4.38 * 10-6
**466**	Enkephalin L	C28H37N5O7	556.27658	Std	Std	Std	Std	Std	Std
**467**	Butirosina	C21H41N5O12	556.28069	2.41 * 10-6	2.03 * 10-6	2.96 * 10-6	4.26 * 10-6	3.29 * 10-6	2.79 * 10-6
**468**		C31H51NOS2	556.30457	2.18 * 10-6	2.61 * 10-6	1.79 * 10-6	2.14 * 10-6	2.00 * 10-6	

**469**	Coroglaucigenin-3-o-alpha-L-rhamnopyranoside	$C_{29}H_{44}O_{9}$	559.28775	3.46 * 10-4	6.34 * 10-2	7.28 * 10-2			
**470**		$C_{28}H_{58}O_{9}$	561.39727	1.11 * 10-7	1.04 * 10-7	8.82 * 10-6	2.38 * 10-6	2.43 * 10-6	2.28 * 10-6
**471**	Linoleic acid dimer	$C_{36}H_{64}O_{4}$	561.48771				3.57 * 10-6	6.90 * 10-6	7.09 * 10-6
**472**		$C_{30}H_{67}N_{5}S_{2}$	562.49097				3.28 * 10-6	5.12 * 10-6	4.61 * 10-6
**473**		$C_{36}H_{66}O_{4}$	563.50337				3.26 * 10-6	6.22 * 10-6	8.02 * 10-6
**474**		$C_{25}H_{33}N_{13}O_{3}$	564.29023				3.57 * 10-6	3.85 * 10-6	3.20 * 10-6
**475**		$C_{24}H_{46}O_{13}$	565.28304	2.95 * 10-7	2.98 * 10-7	2.98 * 10-7	2.53 * 10-7	2.21 * 10-7	2.26 * 10-7
**476**	Oleic acid, dimer	$C_{36}H_{68}O_{4}$	565.51896					3.05 * 10-6	3.67 * 10-6
**477**	Hordatine A	$C_{28}H_{38}N_{8}O_{5}$	567.30114	1.76 * 10-5				1.93 * 10-5	1.64 * 10-5
**478**		$C_{32}H_{61}NO_{7}$	572.45211	3.99 * 10-6	4.09 * 10-6	4.60 * 10-6			
**479**		$C_{26}H_{64}N_{6}O_{3}S_{2}$	573.45528	2.40 * 10-6		1.80 * 10-6			
**480**		$C_{35}H_{63}N_{3}OS$	574.47651	1.74 * 10-6					1.61 * 10-6
**481**		$C_{25}H_{62}N_{6}O_{4}S_{2}$	575.43485					2.06 * 10-6	3.19 * 10-6

#	Putative Annotation[a]	Molecular Formula	Theo. m/z[b]	HA1[c]	HA2[c]	HA3[c]	Org1[c]	Org2[c]	Org3[c]
**482**	Methoxy-hydroxy-all-trans-hexaprenylbenzoate	C38H55O4	576.41606	1.76 * 10-6				1.78 * 10-6	
**483**		C26H46N14	577.39219	1.16 * 10-7	1.34 * 10-7	1.29 * 10-7	8.70 * 10-6	7.93 * 10-6	7.99 * 10-6
**484**		C35H48O5S	581.32975	1.89 * 10-6	3.96 * 10-6	3.13 * 10-6		1.73 * 10-6	
**485**		C19H47N13S4	586.30329				3.19 * 10-6	3.92 * 10-6	3.95 * 10-6
**486**	Chlorophyll c	C35H32N4O5	589.24434		1.96 * 10-5	1.83 * 10-6			
**487**		C21H54N8O5S3	595.34529	1.84 * 10-6	2.46 * 10-6	2.92 * 10-6			
**488**	Proanthocyanidin A2	C30H24O12	599.11600	1.76 * 10-5	3.70 * 10-1	2.78 * 10-2			
**489**		C23H40N5O10P	600.24044	3.60 * 10-7	3.18 * 10-7	2.89 * 10-7			
**490**		C24H63N3O7P2S	600.39344			2.73 * 10-6	2.30 * 10-6		
**491**		C29H44N8S3	601.29242	7.50 * 10-6	6.84 * 10-6	7.20 * 10-6			
**492**		C38H50S3	603.31485	3.08 * 10-6	3.79 * 10-6	3.58 * 10-6			
**493**		C20H60N8O6S3	605.38708	2.17 * 10-6	2.61 * 10-6	2.44 * 10-6			
**494**	Chymostatin	C31H41N7O6	608.31652				2.54 * 10-5	2.53 * 10-6	2.23 * 10-5
**495**	1-hexadecanoyl-2-(4-oxo-6-carboxy-5E-hexenoyl) sn-glycero-3-phosphoethanolamine	C28H50NO11P	608.32072				2.03 * 10-6	3.23 * 10-6	3.02 * 10-6
**496**		C26H50O14	609.30922	2.36 * 10-7	2.46 * 10-7	2.65 * 10-7	1.83 * 10-7	1.78 * 10-7	1.70 * 10-7
**497**	Hydroxyvitamin D2 25-(beta-glucuronide)	C34H52O8	611.35544	1.90 * 10-5					
**498**	_D-Met2, Pro5_-enkephalinamide	C30H40N6O6S	613.28097			1.88 * 10-6	2.14 * 10-6	2.32 * 10-6	2.11 * 10-6
**499**		C26H54O14	613.34053	1.24 * 10-7	1.24 * 10-7	1.31 * 10-7	9.36 * 10-6	8.40 * 10-6	6.36 * 10-6
**500**		C23H43N13OS3	614.29481				1.81 * 10-6		1.67 * 10-6
**501**		C24H55N14O3P	619.43887	3.62 * 10-6	3.14 * 10-6	3.62 * 10-6			1.93 * 10-6
**502**		C14H28N10O10S4	625.09456	7.54 * 10-7	8.20 * 10-7	8.50 * 10-7	1.04 * 10-8	1.05 * 10-8	1.05 * 10-8
**503**	Prunin 6''-O-gallate	C28H26O14	625.09541	7.54 * 10-3					
**504**		C24H17N7O14	628.09084				2.04 * 10-6		1.89 * 10-6
**505**	Peridinin	C39H50O7	631.36293	2.83 * 10-4	2.33 * 10-2				7.24 * 10-2

**506**		C25H52N4O10S2	633.31978	2.66 * 10-6		2.88 * 10-6			
**507**		C38H64O5S	633.45471		2.60 * 10-6	2.80 * 10-6			1.76 * 10-6
**508**		C21H6N4O10S5	634.87590	7.26 * 10-6	4.21 * 10-6	4.39 * 10-6			
**509**	Isaridin F	C34H51N5O7	642.38401				1.93 * 10-6	2.67 * 10-6	2.18 * 10-6
**510**	PE(14:1/15:0(9Z))	C34H66NO8P	648.46206				4.72 * 10-5	5.44 * 10-6	5.43 * 10-5
**511**	Milbemycin alpha10	C37H49NO9	652.34739				2.36 * 10-5	3.24 * 10-6	3.38 * 10-5
**512**	Cer_d(20:1/22:6(5Z,7Z,10Z,13Z,16Z,19Z))	C42H71NO4	654.54369					1.77 * 10-5	1.73 * 10-5
**513**	Oleanoic acid 3-O-glucuronide	C36H56O9	655.38165	1.54 * 10-5					
**514**		C24H70N8O6S3	663.46539	2.32 * 10-6	2.63 * 10-6	2.84 * 10-6	1.84 * 10-6		
**515**		C30H54N14O2	665.44463	5.00 * 10-6	5.80 * 10-6	6.50 * 10-6	4.62 * 10-6	4.40 * 10-6	4.40 * 10-6
**516**		C25H43N5O10P2S	668.22793	6.01 * 10-6	4.23 * 10-6	3.59 * 10-6			
**517**	O-α-D-Galactopyranosylciceritol	C25H44O21	681.24627				1.73 * 10-6		1.66 * 10-6
**518**	PA_12:0_PGF2alpha	C35H63O11P	691.41751		1.79 * 10-6	1.86 * 10-6			

#	Putative Annotation[a]	Molecular Formula	Theo. m/z[b]	HA1[c]	HA2[c]	HA3[c]	Org1[c]	Org2[c]	Org3[c]
**519**		C24H60N12O9S	693.43991		1.71 * 10-6	2.27 * 10-6			
**520**	HMBOA dihexose	C27H39NO20	698.21730	3.43 * 10-6	4.02 * 10-6	3.24 * 10-6			
**521**	Leucomycin	C35H59NO13	702.40851	2.17 * 10-6	2.58 * 10-6				1.84 * 10-6
**522**	PE(14:1(9Z)/20:4(5Z,8Z,11Z,14Z))	C39H68NO8P	710.47399	2.13 * 10-6	1.92 * 10-6				
**523**	Strophanthin	C36H54O14	711.35770	2.09 * 10-6	2.97 * 10-6	2.14 * 10-6			
**524**	PA(16:0/PGF1alpha)	C39H73O11P	749.49660	1.63 * 10-6				1.62 * 10-5	
**525**	DG_22:0_PGF2alpha_0:0_	C45H82O8	751.60888				1.62 * 10-6		1.54 * 10-6
**526**	PC(18:0/20:3(5Z,8Z,11Z))	C46H84NO9P	826.59452		2.43 * 10-6	2.62 * 10-5			

a Cer Ceramide; DG Diacylglycerol; MG Monoacylglycerol; PA Phosphatidic acid; PC Phosphatidylcholine; PE Phosphatidylethanolamine; PG Glycerophospholipids; PGE2 Prostaglandin E2; PI Phosphatidylinositol

b Theoretical mass

c HA stands for hydroalcoholic extract and Org stands for organic extract.
